# Unusual Racemization of Tertiary *P*‐Chiral Ferrocenyl Phosphines

**DOI:** 10.1002/chem.202000218

**Published:** 2020-04-01

**Authors:** John Popp, Schirin Hanf, Evamarie Hey‐Hawkins

**Affiliations:** ^1^ Faculty of Chemistry and Mineralogy Institute of Inorganic Chemistry Leipzig University Johannisallee 29 04103 Leipzig Germany; ^2^ Department of Chemistry Cambridge University Lensfield Road Cambridge CB2 1EW UK

**Keywords:** chirality, ferrocene, inversion, phosphines, racemization

## Abstract

Tertiary phosphines are generally known to withstand inversion under moderate conditions. In this work, a remarkable racemization process of three *P*‐chiral ferrocenyl phosphines is reported. Subjected to conventional column chromatography as highly enantioenriched compounds, they greatly experienced racemization when collected at the column outlet within minutes. Initially, attention was drawn to this unusual inversion behavior after observing that the superb enantiomeric excess of these ligands (>95 % *ee* in all cases) was almost lost in their corresponding ruthenium(II) complexes. Successively excluding possible racemization causes, these *P*‐chiral ferrocenyl phosphines were found to undergo a significant, acid‐catalyzed racemization process at room temperature within a few minutes. This process is mainly observed during standard column chromatography by using conventional silica or alumina, but can also be triggered deliberately by addition of certain acids. Therefore, the stereochemical preservation of *P*‐chiral phosphines during their purification may per se not always be guaranteed, since column chromatography is the most frequently used technique for purifying such types of compounds.

## Graphical abstract

**Enantiomeric excess vanished**! Subjected to conventional column chromatography as highly enantioenriched compounds, *P*‐chiral ferrocenyl phosphines suffered dramatically from racemization when collected at the column outlet even within minutes. What has happened? The authors have thoroughly investigated this most frequently used technique for the purification of such compounds and found a remarkable process as the explanation.

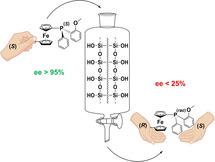

The basic concept that a phosphorus atom with three different substituents gives rise to a pair of configurationally stable enantiomers has been discussed since the beginning of the 20th century.^1^ Starting from simple studies about phosphorus stereochemistry, methods for the preparation of “tailor‐made” optically pure *P*‐stereogenic compounds as versatile ligands in asymmetric homogeneous catalysis were developed.^2^, ^3^, ^4^, ^5^ In particular, the early studies on enantioselective hydrogenations utilizing *P*‐stereogenic phosphine ligands pushed active research in this field forward.^6^, ^7^ Despite the successful commercialization of DIPAMP^8^ for the industrial production of l‐DOPA,^9^ subsequent research within the class of *P*‐stereogenic phosphines suffered from synthetic difficulties, leading to a rising number of publications on backbone‐chiral phosphines, such as DIOP,^10^ CHIRAPHOS,^11^ and BINAP.^12^ In this regard, ferrocene‐based^13^ chiral phosphine ligands designed for asymmetric synthesis have attracted tremendous scientific interest over the past decades;^14^ some have also been applied successfully in asymmetric hydrogenation on an industrial level.^15^, ^16^ Furthermore, similar ligand systems utilizing multiple elements of chirality were developed showing superb enantioselectivities in iridium‐17 and rhodium‐catalyzed^18^ hydrogenations.

Our research on new classes of ferrocenyl phosphines has shifted from aryl‐based ferrocenyl phosphine ligands for rhodium(I)‐catalyzed hydroformylation of olefins^19^, ^20^ to immobilized ferrocenyl phosphine ligands for ruthenium(II)‐catalyzed isomerization of allylic alcohols.^21^ This first example of redox‐switchable catalysis involving dendritic phosphine‐containing ligands^22^ encouraged us to introduce *P*‐stereogenic ferrocenyl phosphines into the ligand system opening the door to asymmetric catalysis.

The *P*‐stereogenic ferrocenyl phosphine boranes **1 a**–**c** 23 were successfully synthesized from an unsymmetrically disubstituted 1,1′‐ferrocene^22^ and three methyl (phenyl)phosphinite boranes (Scheme 1) which were accessible via an (−)‐ephedrine‐based oxazaphospholidine borane complex.^24^, ^25^ This auxiliary‐based methodology provides a convenient strategy for a stepwise preparation of tertiary *P*‐stereogenic phosphines (>95 % *ee* in all cases). In contrast to similarly prepared monodentate^26^ or bidentate^27^, ^28^ ligands, **1 a**–**c** contain a 4‐methoxyphenyl substituent in the 1′‐position of the ferrocenyl group. Replacement of 4‐methoxyphenyl in these model complexes with 4‐hydroxyphenyl allows for immobilization of these ligands on a variety of solid supports.

**Scheme 1:**
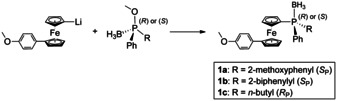
*P*‐Stereogenic ferrocenyl phosphine boranes **1 a**–**c**.

The first indications that made us investigate the phosphorus‐based stereochemistry of these ligands in detail were the single crystal X‐ray crystallographic analyses of their corresponding ruthenium(II) complexes **3 a**–**c** 23 (Figure 1). In repeated attempts, we continuously found that complexes **3 a** and **3 b** crystallize in the achiral space group *P* $\bar{1}$ indicating the presence of racemates. In contrast to the borane‐protected ligands **1 a**–**c**, the determination of the enantiomeric excess of the corresponding ruthenium(II) complexes **3 a**–**c** by chiral HPLC was not successful. The results of the screening on several phases in different screening modes were inconclusive; no isomer selectivity was observed. In order to gain information about the loss of enantiopurity upon complexation, we carried out decomplexation experiments using the more electron‐rich triethyl phosphine for replacing the ferrocenyl phosphine ligand in **3 a**–**c** (Scheme 2). After borane‐protection of the resulting free phosphines **2 a**–**c**, HPLC analysis of **1 a**–**c** revealed a dramatic decrease of the enantiomeric excess requiring an in‐depth stereochemical elucidation.

**Figure 1:**
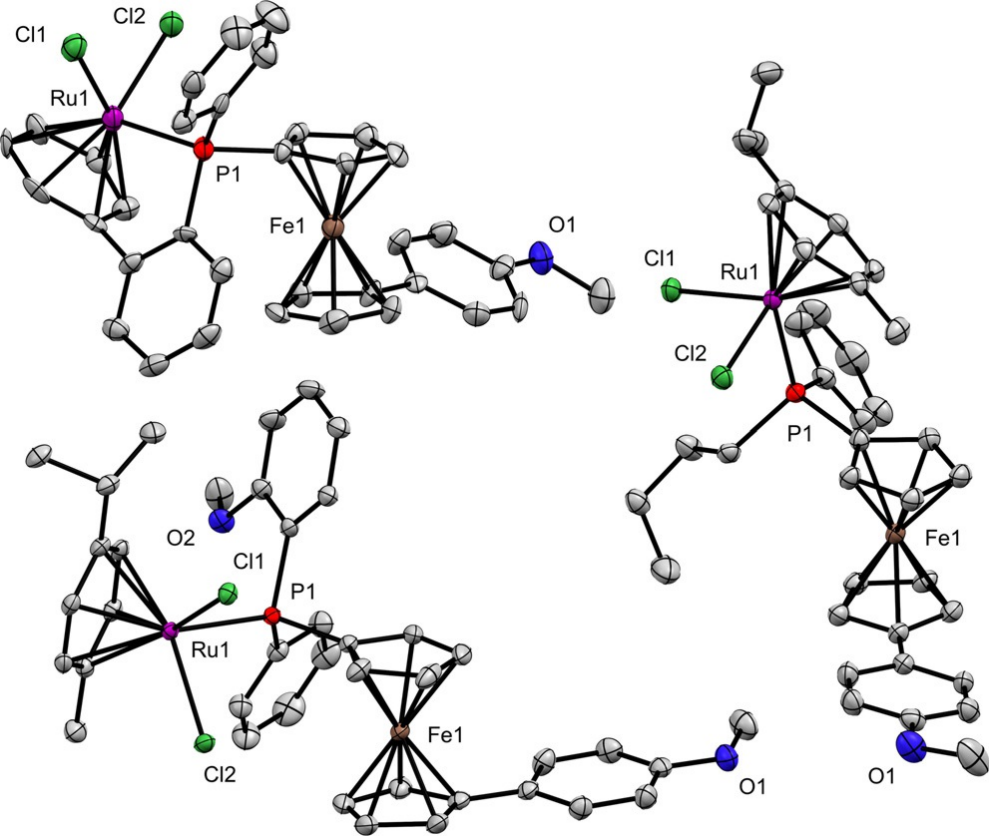
Molecular structure of **3 b** 23 (top left, *P* $\bar{1}$ ), **3 a** 23 (bottom left, *P* $\bar{1}$ ) and **3 c** (right, *P*2_1_) in the solid state. Ellipsoids at 50 % probability; hydrogen atoms are omitted for clarity.

**Scheme 2:**
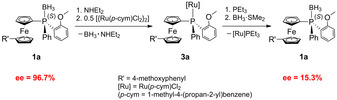
Complexation–decomplexation sequence shown exemplarily for **1 a**.

In order to rule out the influence of the borane‐protection/deprotection step on the enantiopurity of the ligands, the BH_3_ group in **1 a**–**c** was removed by heating in the presence of diethylamine; thereby, the free phosphines **2 a**–**c** were obtained in excellent yields. In order to determine their enantiomeric excesses reliably, reprotection with BH_3_ and subsequent measurement via HPLC was performed. A comparison of the enantiomeric excesses of **1 a**–**c** before and after the deprotection‐reprotection sequence confirmed the preservation of stereochemistry (differences within tolerance limits) eliminating the borane‐protecting step as a possible racemization cause.

To investigate whether the observed racemization is temperature dependent, thermal inversion barriers of ferrocenyl phosphines **2 a**–**c** had to be determined. Inspired by significant research on the configurational lability of *P*‐stereogenic phosphines,^29^, ^30^, ^31^, ^32^, ^33^ we determined inversion barriers of **2 a**–**c** experimentally by heating in toluene, following the decay of the enantiomeric excess over time, and supported the results by DFT calculations (Table 1, see the Supporting Information for details). All values are above 125 kJ mol^−1^, which is in the expected range for trivalent aryl phosphorus compounds,^31^, ^34^, ^35^, ^36^ and the DFT calculations are in excellent agreement with the experimental values implying that a thermally induced racemization cannot explain the observed racemization.

**Table 1:** Inversion barriers of **2 a**–**c** in kJ mol^−1^.

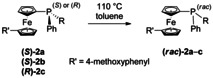			
R	exptl^[a]^	calcd^[b]^	Δ
2‐methoxyphenyl (**2 a**)	125.1	125.4	0.3
2‐biphenylyl (**2 b**)	126.5	136.6	10.1
*n*‐butyl (**2 c**)	132.7	136.5	3.8

All given values are in kJ mol^−1^. [a] See the Supporting Information for details. [b] DFT with ORCA:^37^, ^38^ PWPB95^39^/def2‐QZVP^40^, ^41^.

Upon pyramidal inversion, trivalent group 15 compounds generally undergo structural and electronic changes^42^, ^43^ which are frequently characterized within the context of a second‐order Jahn–Teller effect.^44^, ^45^, ^46^, ^47^ Furthermore, their racemization can be triggered by electronic alteration. As previously evidenced,^29^ tertiary ferrocenyl phosphines can undergo an outer‐sphere single electron transfer when treated with a suitable oxidizing agent leading to a rapid pyramidal inversion at ambient temperatures. An electron transfer from the ferrocenyl phosphine to ferrocenium hexafluorophosphate generates a transient oxidized species for which racemization via a low‐energy pyramidal inversion pathway is preferred over a return electron transfer.^29^ Our study of the optical behavior of *P*‐stereogenic ferrocenyl phosphines upon single‐electron oxidation supported the earlier findings and, furthermore, broadened the scope of ferrocenyl phosphines for which this observation applies. By adding 25 mol % ferrocenium hexafluorophosphate, the enantiomeric excesses of **2 a**–**c** dropped below 10 % in all cases within 30 min at room temperature (Table 2). Additionally, we computed inversion barriers of the oxidized ferrocenyl phosphines by DFT methods in order to endorse the experimental observations. Overall, significant decreases of the computed inversion barriers were found for **2 a**–**c**, meaning that their oxidized transition state inversion barriers dropped to 64.2, 91.6 and 80.6 kJ mol^−1^ for **2 a**, **2 b** and **2 c**, respectively (see Table 1 for corresponding neutral ground state values).

**Table 2:** Racemization of **2 a**–**c** catalyzed by single‐electron oxidation using ferrocenium hexafluorophosphate. All given values are in %.

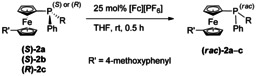			
R	Initial *ee*	Final *ee* ^[a]^	Reference *ee* ^[b]^
2‐methoxyphenyl (**2 a**)	97.2	9.3	97.1
2‐biphenylyl (**2 b**)	98.9	0	98.9
*n*‐butyl (**2 c**)	95.0	4.6	94.7

[a] See the Supporting Information for details. [b] Reference experiments were performed simultaneously without adding ferrocenium hexafluorophosphate.

In comparison, aryl‐based trivalent *P*‐stereogenic phosphines which do not contain a ferrocenyl substituent revealed pyramidal inversion barriers for those single oxidized phosphines of around 20 kJ mol^−1^ in comparable experiments.^29^ Since the structural deformation towards a planar transition state is associated with raising of the HOMO energy due to loss of HOMO–LUMO orbital mixing,^48^ it appears that the ferrocenyl substituent is counteracting this energetic penalty. The structural flattening of the ground state towards a relaxed pyramidal geometry as a reaction of the removal of one electron is expressed by the reduction of the internal dihedral angle. For instance, the PCCC dihedral angle of methylphenyl(2‐methylphenyl)phosphine is reduced by 17° upon single‐electron oxidation.^29^ In contrast, we found that for the geometrically optimized structures of ferrocenyl phosphines **2 a**–**c** this dihedral angle was decreased only marginally (0.3°, 1.5° and 0.6° for **2 a**, **2 b** and **2 c**, respectively). Apparently, the ferrocenyl substituent plays a major role for electronically stabilizing the single oxidized phosphines so that a noticeable structural change is not favorable, as seen for trivalent *P*‐stereogenic phosphines which do not contain a ferrocenyl substituent. Interestingly, although there is almost no structural flattening occurring upon oxidation the transition state inversion barriers are still significantly lowered compared to the corresponding neutral ground states.

A key experiment in the explanation of the racemization mechanism was time‐resolved column chromatography of the *P*‐stereogenic ferrocenyl phosphines **2 a**–**c**. Having other possible triggers for racemization excluded, the enantiomeric excess of the free phosphines was monitored while running conventional column chromatography. Fractions of the sample were collected at certain times, and a remarkable decrease of the enantiomeric excess within minutes was observed (Figure 2, left). Regardless of the substitution at the phosphorus atom, **2 a**–**c** show a highly similar decay of their enantiomeric excess. After 60 minutes on silica, the enantiomeric excess has dropped below 25 % in all cases. The remaining enantiomeric excess of **2 a**–**c** at 60 minutes qualitatively resembles their experimentally determined and calculated thermal inversion barrier; for example, **2 c** has the highest inversion barrier and also racemizes the slowest during chromatography using silica. The estimated half‐life of the enantiopurity of these ferrocenyl phosphines is 25 minutes, which is not explicable by using the classical thermal racemization mechanism for trivalent *P*‐stereogenic phosphines.

**Figure 2:**
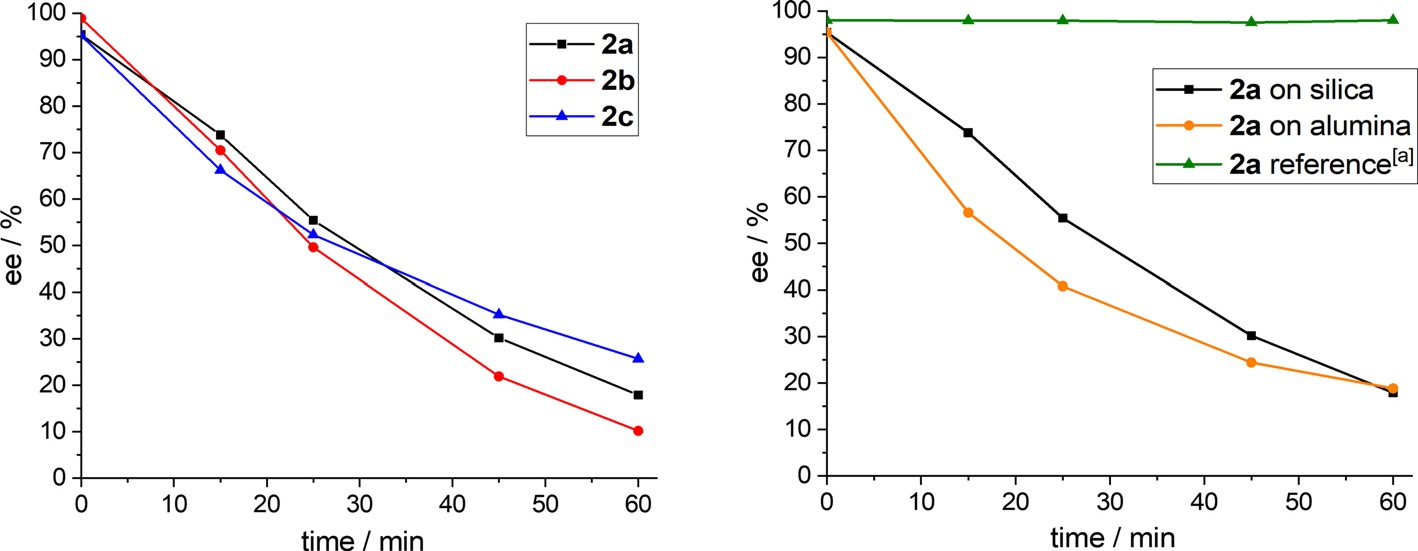
(Left) Decay of enantiomeric excess of **2 a**–**c** over time during column chromatography on silica. (Right) Decay of enantiomeric excess of **2 a** over time on silica and on alumina. [a] Reference experiment was performed without using silica or alumina, no solid stationary phase was used.

Extending the scope, we repeated the time‐resolved chromatographic experiment with **2 a** by using alumina as solid stationary phase for column chromatography. A highly similar outcome was found by showing that also with alumina the enantiomeric excess of **2 a** drops to 20 % after a contact time of 60 minutes with the stationary phase (Figure 2, right). Moreover, we utilized a rather unconventional approach revealing the solid stationary phase as the only possible racemization cause, where we repeated the experiment without using any stationary phase at all. Keeping all other experimental details identical, **2 a** did not show any signs of racemization over the course of 60 minutes (Figure 2, right, reference). To prove that the observed racemization is solely caused by the acidic surface of silica and alumina, the experiments were repeated after pre‐treatment of the stationary phase with 5 % triethylamine in hexane, a common technique for purifying acid‐sensitive compounds. Indeed, the deactivation of the surface silanol groups prevented racemization completely. Furthermore, the use of basic alumina protects the enantiopurity of the *P*‐stereogenic ferrocenyl phosphines.

Unfortunately, attempts to mimic the acidic silanol groups of the silica surface by using a trisilanol‐isooctyl‐substituted polyhedral silsesquioxane (PSS) in dichloromethane were repeatedly not successful. Being well‐defined molecular compounds with incompletely condensed open‐cage structures, silsesquioxanes are commonly used to model the surface of silica.^49^ Molecular properties of amorphous silica are strongly affected by their surface hydroxyl functionalities, which exist as vicinal, geminal or isolated silanol sites. PSS are generally not able to model geminal silanols^50^ which might explain why **2 a**–**c** did not show any signs of racemization in the silica‐mimicking experiments. Depending on concentration and solvent, PSS, furthermore, form dimers in solution lowering their acidity.^51^, ^52^ In this regard, the use of silanetriols might be better suited to model the acidic surface of silica.^53^

Recapitulating all reported experiments, we concluded that the initially observed racemization is caused by an acid‐catalyzed process induced by silica and alumina. In order to further substantiate our conclusion, we tested the racemization behavior of **2 a** under acidic conditions. Tetrafluoroboric acid or trifluoroacetic acid led to complete racemization of **2 a** in dichloromethane within 30 minutes (*ee*<1 %). Treatment with diluted hydrochloric acid and phosphoric acid showed only minor racemization after 30 minutes (90.9 % and 92.5 %, respectively). Stronger trifluoromethanesulfonic acid, resulted in irreversible oxidation of the ferrocenyl phosphine. DFT calculations^37^, ^38^, ^39^, ^40^, ^41^ on the structure of protonated **2 a** gave a hint on how the pyramidal inversion leading to racemization is triggered (Figure 3). By protonating the phosphine, the internal dihedral angle (PCCC) decreased by 14.6°, and a decreased PCCC angle leads to lowering of the corresponding inversion barrier. An in‐depth elucidation on how the actual inversion from this initial protonated compound takes place is part of our ongoing research on *P*‐stereogenic ferrocenyl phosphines.

**Figure 3:**
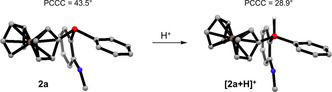
Calculated^37^, ^38^, ^39^, ^40^, ^41^ structure of **2 a**. Upon protonation the internal dihedral angle (PCCC) is significantly decreased. Hydrogen atoms, except for phosphorus‐bound proton, and 4‐methoxyphenyl groups are omitted for clarity.

In summary, an unexpected racemization of three *P*‐stereogenic ferrocenyl phosphines was observed during the course of conventional chromatography on silica or alumina. While these phosphines are configurationally stable on heating, they undergo an acid‐catalyzed racemization at room temperature within a few minutes. Our research highlights that stereochemical preservation of *P*‐stereogenic phosphines during purification processes may per se not always be guaranteed.

## Experimental Section

**Crystallographic data**: CCDC 1948526 (**1c**) and 1948527 (**3c**) contain the supplementary crystallographic data for this paper. These data are provided free of charge by The Cambridge Crystallographic Data Centre.

## Conflict of interest

The authors declare no conflict of interest.

## Supporting information

As a service to our authors and readers, this journal provides supporting information supplied by the authors. Such materials are peer reviewed and may be re‐organized for online delivery, but are not copy‐edited or typeset. Technical support issues arising from supporting information (other than missing files) should be addressed to the authors.

Supplementary
