# Supplementary material for: Unusual Racemization of Tertiary P‐Chiral Ferrocenyl Phosphines
Source: Chemistry. 2020 Apr 1;26(26):5765–9. doi: 10.1002/chem.202000218 (PMC7317868; doi:10.1002/chem.202000218)
Supplement: Supplementary file 1 — Supplementary [file CHEM-26-5765-s001.pdf]

Chemistry–A European Journal

Supporting Information

Unusual Racemization of Tertiary *P*-Chiral Ferrocenyl Phosphines

John Popp,[a] Schirin Hanf,[a, b] and Evamarie Hey-Hawkins*[a]

Table of Contents

General Procedures and Analytical Methods S2
Synthesis of Ferrocenyl Phosphine Borane **1c** S3
Synthesis of Ferrocenyl Phosphine **2c** S3
Synthesis of Ferrocenyl Phosphine Ruthenium(II) Complex **3c** S4
Decomplexation Reaction S5
Deprotection-Reprotection Reaction Sequence S5
Racemization of Ferrocenyl Phosphines S6
X-ray Crystallography S8
Computational Details S9
HPLC Chromatograms S30
NMR Spectra S32
References S41

General Procedures and Analytical Methods

In case of moisture or air sensitivity, the reactions were conducted under nitrogen atmosphere using Schlenk techniques. Diethyl ether, dichloromethane, toluene and hexane (isomeric mixture) were obtained from an MBraun Solvent Purification System SPS-800 and stored over 4 Å molecular sieves or potassium (in case of hexane). THF was dried and distilled from potassium, diethylamine was dried and distilled from potassium hydroxide. The starting material 1-bromo-1'-(4-methoxyphenyl)ferrocene[1] was synthesized from 1,1'-dibromoferrocene[2] according to literature procedures. The methyl (phenyl)phosphinite boranes[3] were synthesized via an (–)-ephedrine-based oxazaphospholidine borane complex. We have previously reported the synthesis and characterization of **1a,b**, **2a,b** and **3a,b**.[4] Ferrocenium hexafluorophosphate was synthesized by oxidation of ferrocene with concentrated sulfuric acid and subsequent treatment with ammonium hexafluorophosphate. Di-µ-chlorobis-[(η^6-*p*-cymene)chlororuthenium(II)] is commercially available and was used without further purification. NMR spectra were recorded with a Bruker Avance III HD 400 or Bruker Ascend 400 spectrometer. Mass spectra were obtained with a Bruker ESI-TOF micrOTOF, a Bruker ESI-qTOF Impact II and a Bruker Esquire 3000plus spectrometer. Elemental analyses were determined with a Heraeus Vario EL Analyzer. IR spectra were obtained with a Perkin-Elmer FT-IR Spectrum 2000 spectrometer. The samples were measured as KBr pellets. Chiral HPLC was performed with a Knauer HPLC system with a Smartline PDA 2800 detector (λ = 233 nm) and a 250 x 4.6 mm Lux® 5 µm Amylose-1 column by Phenomenex. Common column chromatography was performed using Merck Geduran Silicagel 60 (40–63 µm) and aluminum oxide (activated, neutral or basic, Bockmann Activity I) from Sigma-Aldrich. The specific rotations were measured with a Krüss Optronic P3002RS automatic digital polarimeter using a 1 dm micro polarimeter tube from Schmidt + Haensch. Melting points were determined using a Gallenkamp MPD 350 BM 2.5 capillary melting point apparatus and are reported uncorrected.

Synthesis of Ferrocenyl Phosphine Borane 1c

1-[(R_P)-(*n*-Butyl)(phenyl)phosphine *P*-borane]-1'-(4-methoxyphenyl)ferrocene

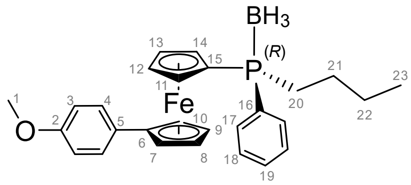

At –80 °C, ^{n}BuLi in hexanes (1.10 eq.) was slowly added to a solution of 1-bromo-1'-(4-methoxyphenyl)ferrocene (1.05 eq.) in THF (0.25 mol·L^{-1}). After stirring for 1 h at –80 °C, the reaction mixture was slowly added to a solution of methyl (S_P)-(*n*-butyl)(phenyl)-phosphinite *P*-borane (1.00 eq., 0.71 g, 3.38 mmol) in THF (0.25 mol·L^{-1}) at –80 °C. The solution was allowed to warm to room temperature over a period of 12 h. Water was added and the aqueous phase was extracted with diethyl ether. The combined organic layers were washed with saturated aqueous NaCl and dried over $MgSO_4$. After removal of the solvent in vacuum, the crude product was purified by column chromatography on silica with hexane/DCM 4:1 grad. hexane/DCM 1:1 (1.09 g, 68%). Its enantiomeric excess was determined by analytical chiral HPLC (hexane/isopropanol 90:10, 1.0 ml·min^{-1}): ee = 95.6% (t_R: 7.4 min [S_P], 9.0 min [R_P]).

R$_f$: 0.48 (hexane/DCM 2:1, v/v)
$[\alpha]_D^{25}$: +68.1° (*c* 1.25, $CHCl_3$)
^{1}H-NMR (400.2 MHz, $CDCl_3$): δ (ppm) = 0.61–1.67 (br m, 3H, BH_3), 0.83–0.86 (m, 3H, H23), 1.24–1.34 (m, 2H, H22), 1.49–1.58 (m, 2H, H21), 1.92–1.99 (m, 2H, H20), 3.81 (s, 3H, H1), 4.19–4.21 (m, 2H, H11/H14, H12/H13), 4.26–4.30 (m, 3H, H8, H9, H11/H14), 4.34–4.35 (m, 1H, H12/H13), 4.49–4.51 (m, 1H, H7/H10), 4.67–4.68 (m, 1H, H7/H10), 6.81 (d, 2H, $^3J_{HH}$ = 8.5 Hz, H4), 7.30 (d, 2H, $^3J_{HH}$ = 8.6 Hz, H3), 7.39–7.54 (m, 3H, H18, H19), 7.69–7.79 (m, 2H, H17)
^{13}C{^{1}H}-NMR (100.6 MHz, $CDCl_3$): δ (ppm) = 13.7 (s, C23), 24.3 (d, $^2J_{CP}$ = 14.4 Hz, C21), 25.4 (s, C22), 27.6 (d, $^1J_{CP}$ = 38.9 Hz, C20), 55.4 (s, C1), 67.4 (s, C7/C10), 67.5 (s, C7/C10), 70.7 (s, C8/C9), 70.8 (s, C8/C9), 71.0 (d, $^1J_{CP}$ = 65.4 Hz, C15), 72.9 (d, $^3J_{CP}$ = 9.5 Hz, C12/13), 73.2 (d, $^3J_{CP}$ = 9.7 Hz, C12/13), 73.5 (d, $^2J_{CP}$ = 7.4 Hz, C11/C14), 73.9 (d, $^2J_{CP}$ = 7.5 Hz, C11/C14), 87.2 (s, C6), 114.0 (s, C4), 127.3 (s, C3), 128.6 (d, $^3J_{CP}$ = 9.7 Hz, C18), 130.0 (s, C5), 130.5 (d, $^1J_{CP}$ = 55.0 Hz, C16), 131.1 (d, $^4J_{CP}$ = 2.1 Hz, C19), 132.2 (d, $^2J_{CP}$ = 8.9 Hz, C17), 158.4 (s, C2)
^{31}P{^{1}H}-NMR (162.0 MHz, $CDCl_3$): δ (ppm) = 11.4 (br m)
ESI(+)-MS: *m/z* (%) = 493.2 (37.8) [M+Na]$^+$, 470.2 (100) [M]$^+$
FT-IR (KBr): $\tilde{\nu}$ (cm^{-1}) = 3438 w, 3080 w, 3055 w, 2997 w, 2857 m, 2931 m, 2868 w, 2834 w, 2378 m, 2343 m, 2254 w, 2037 w, 1967 w, 1890 w, 1610 m, 1576 w, 1526 s, 1483 w, 1459 s, 1438 m, 1384 w, 1341 w, 1305 m, 1288 m, 1248 s, 1210 w, 1176 s, 1135 w, 1109 m, 1065 m, 1031 s, 889 w, 865 m, 830 s, 743 m, 697 m, 651 w, 630 w, 603 m, 533 m, 494 m, 463 m, 419 w
HR-MS (ESI(+)): *m/z* [M]$^+$ calcd for $C_{27}H_{32}BFeOP$: 470.1633; found: 470.1627

Synthesis of Ferrocenyl Phosphine 2c

1-[(R_P)-(*n*-Butyl)(phenyl)phosphine]-1'-(4-methoxyphenyl)ferrocene

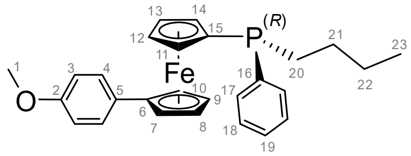

A solution of **1c** (0.75 g, 1.58 mmol) in diethylamine (0.025 mol·L^{-1}) was heated at 50 °C for 12 h. Diethylamine was removed by evaporation under reduced pressure and the crude residue was purified by column chromatography on degassed, deactivated silica (pretreated with hexane/triethylamine 95:5) with degassed hexane/DCM 2:1 grad. hexane/DCM 1:2 (0.70 g, 97%). In order to determine the enantiomeric excess, **2c** was reprotected by reaction with BH_3·SMe_2 (2.0 mol·L^{-1} in THF) prior analysis by analytical chiral HPLC (hexane/isopropanol 90:10, 1.0 ml·min^{-1}): ee = 95.0% (t_R: 7.4 min [S_P], 8.9 min [R_P]).
R$_f$: 0.51 (hexane/DCM 2:1, v/v)
mp: 36–38 °C
^{1}H-NMR (400.2 MHz, CD_2Cl_2): δ (ppm) = 0.87–0.98 (m, 3H, H23), 1.21–1.57 (m, 4H, H21, H22), 1.81–1.93 (m, 2H, H20), 3.81 (s, 3H, H1), 3.96–3.98 (m, 1H, H11/H14), 4.13–4.15 (m, 1H, H12/H13), 4.16–4.17 (m, 1H, H11/H14), 4.20–4.21 (m, 1H, H12/H13), 4.25–4.26

(m, 2H, H8, H9), 4.54–4.55 (m, 1H, H7/H10), 4.58–4.60 (m, 1H, H7/H10), 6.85 (d, 2H, $^3J_{HH}$ = 8.7 Hz, H4), 7.26–7.33 (m, 3H, H18, H19), 7.42 (d, 2H, $^3J_{HH}$ = 8.7 Hz, H3), 7.41–7.47 (m, 2H, H17)
^{13}C{^{1}H}-NMR (100.6 MHz, $CDCl_3$): δ (ppm) = 14.0 (s, C23), 24.5 (d, $^2J_{CP}$ = 13.3 Hz, C21), 28.6 (d, $^3J_{CP}$ = 7.7 Hz, C22), 28.7 (d, $^1J_{CP}$ = 23.1 Hz, C20), 55.4 (s, C1), 67.1 (s, C7/C10), 67.2 (s, C7/C10), 69.8 (s, C8/C9), 69.9 (s, C8/C9), 71.8 (d, $^3J_{CP}$ = 7.7 Hz, C12/13), 72.1 (d, $^3J_{CP}$ = 1.9 Hz, C12/13), 72.6 (d, $^2J_{CP}$ = 4.7 Hz, C11/C14), 74.4 (d, $^2J_{CP}$ = 19.3 Hz, C11/C14), 78.3 (d, $^1J_{CP}$ = 8.1 Hz, C15), 86.6 (s, C6), 114.0 (s, C4), 127.4 (s, C3), 128.2 (d, $^3J_{CP}$ = 7.1 Hz, C18), 128.7 (s, C19), 130.8 (s, C5), 132.9 (d, $^2J_{CP}$ = 19.6 Hz, C17), 140.1 (d, $^1J_{CP}$ = 13.7 Hz, C16), 158.3 (s, C2)
^{31}P{^{1}H}-NMR (162.0 MHz, CD_2Cl_2): δ (ppm) = –28.7 (s)
ESI(+)-MS: *m/z* (%) = 487.1 (92.5) $[M+MeOH]^+$, 457.1 (100) $[M+H]^+$
FT-IR (KBr): ṽ (cm^{-1}) = 3436 m, 3069 w, 3046 w, 3005 w, 2958 m, 2925 m, 2854 m, 2212 w, 2036 w, 1946 w, 1886 w, 1741 w, 1642 w, 1610 m, 1579 w, 1527 s, 1458 s, 1433 m, 1378 w, 1340 w, 1304 w, 1289 w, 1252 s, 1178 s, 1158 m, 1109 m, 1086 m, 1065 w, 1029 s, 968 w, 887 w, 854 w, 826 s, 741 s, 694 s, 646 w, 631 w, 605 w, 528 m, 502 s, 482 m, 457 w
Anal. Calcd. for $C_{27}H_{29}FeOP$ (456.1): C 71.06, H 6.41; found: C 71.19, H 6.34

Synthesis of Ferrocenyl Phosphine Ruthenium(II) Complex 3c

1-[(*R*P)-(*n*-Butyl)(phenyl)phosphino][(η^6-*p*-cymene)dichlororuthenium(II)]-1'-(4-methoxyphenyl)ferrocene

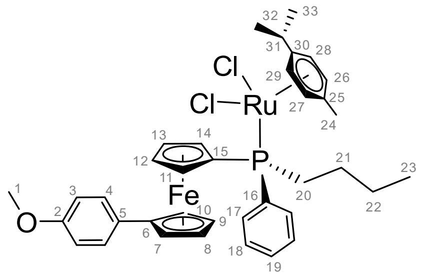

A solution of di-µ-chlorobis-[(η^6-*p*-cymene)chlororuthenium(II)] (0.50 eq.) in DCM (0.025 mol·L^{-1}) was added to a solution of **2c** (1.00 eq., 0.30 g, 0.66 mmol) in DCM (0.025 mol·L^{-1}) and stirred at room temperature until the ^{31}P{^{1}H}-NMR spectrum indicated full conversion of the free phosphine. The volume of the reaction mixture was reduced under reduced pressure, and the resulting residue was purified by column chromatography on degassed silica with degassed pure DCM grad. DCM/ethyl acetate 1:1. Redissolving in DCM (3 mL) and precipitation with hexane (100 mL) gave an orange solid (0.48 g, 95%). The determination of the enantiomeric excess of **3c** was not successful. The results of the screening on a number of phases in different screening modes were inconclusive; no isomer selectivity was observed (chiral HPLC screening service by Phenomenex).

R_f: 0.46 (DCM/ethyl acetate 8:1, v/v)
mp*:* 105–107 °C
^{1}H-NMR (400.2 MHz, $CDCl_3$): δ (ppm) = 0.82 (t, 3H, $^3J_{HH}$ = 7.2 Hz, H23), 0.96 (d, 3H, $^3J_{HH}$ = 6.9 Hz, H32/H33), 1.00 (d, 3H, $^3J_{HH}$ = 6.8 Hz, H32/H33), 1.18–1.35 (m, 4H, H21, H22), 1.58 (s, 3H, H24), 2.26–2.45 (m, 2H, H20), 2.83–2.93 (m, 1H, H31), 3.84 (s, 3H, H1), 4.26–4.28 (m, 1H, H12/H13), 4.30–4.31 (m, 1H, H11/H14), 4.32–4.34 (m, 1H, H12/H13), 4.36–4.39 (m, 2H, H8, H9), 4.54–4.56 (m, 1H, H11/H14), 4.65–4.67 (m, 1H, H7/H10), 4.71–4.72 (m, 1H, H7/H10), 4.94 (d, 1H, $^3J_{HH}$ = 5.9 Hz, H28/H29), 4.99 (d, 1H, $^3J_{HH}$ = 5.7 Hz, H26/H27), 5.09 (d, 1H, $^3J_{HH}$ = 6.0 Hz, H26/H27), 5.17 (d, 1H, $^3J_{HH}$ = 6.0 Hz, H28/H29), 6.89 (d, 2H, $^3J_{HH}$ = 8.7 Hz, H4), 7.37–7.46 (m, 3H, H18, H19), 7.44 (d, 2H, $^3J_{HH}$ = 8.7 Hz, H3), 8.12–8.17 (m, 2H, H17)
^{13}C{^{1}H}-NMR (100.6 MHz, $CDCl_3$): δ (ppm) = 14.1 (s, C23), 17.3 (s, C24), 21.7 (s, C32/C33), 22.6 (s, C32/C33), 24.4 (d, $^2J_{CP}$ = 13.2 Hz, C21), 25.2 (d, $^1J_{CP}$ = 30.4 Hz, C20), 26.8 (d, $^3J_{CP}$ = 8.3 Hz, C22), 30.2 (s, C31), 55.5 (s, C1), 67.7 (s, C7/C10), 68.1 (s, C7/C10), 71.5 (s, C8/C9), 71.8 (s, C8/C9), 73.4 (d, $^3J_{CP}$ = 8.4 Hz, C12/13), 73.5 (d, $^2J_{CP}$ = 6.3 Hz, C11/14), 73.9 (d, $^3J_{CP}$ = 6.8 Hz, C12/13), 74.6 (d, $^2J_{CP}$ = 13.6 Hz, C11/14), 77.7 (d, $^1J_{CP}$ = 44.8 Hz, C15), 83.9 (d, $^2J_{CP}$ = 3.8 Hz, C28/C29), 86.0 (d, $^2J_{CP}$ = 7.2 Hz, C28/C29), 87.5 (s, C6), 88.4 (d, $^2J_{CP}$ = 4.1 Hz, C26/C27), 90.3 (d, $^2J_{CP}$ = 3.4 Hz, C26/C27), 94.5 (s, C25), 108.8 (s, C30), 114.3 (s, C4), 127.6 (s, C3), 128.1 (d, $^3J_{CP}$ = 9.5 Hz, C18), 129.8 (s, C5), 130.1 (d, $^4J_{CP}$ = 2.2 Hz, C19), 132.4 (d, $^2J_{CP}$ = 8.6 Hz, C17), 134.9 (d, $^1J_{CP}$ = 41.8 Hz, C16), 158.7 (s, C2)
^{31}P{^{1}H}-NMR (162.0 MHz, $CDCl_3$): δ (ppm) = 15.3 (s)
ESI(+)-MS: *m/z* (%) = 727.2 (100) $[M–Cl]^+$, 465.2 (37.1) [M–Ru(η^6-*p*-cymene)$Cl_2]^+$
FT-IR (KBr): ṽ (cm^{-1}) = 3444 w, 3074 w, 2957 m, 2928 m, 2868 m, 2834 w, 2036 w, 1733 w, 1609 m, 1575 w, 1525 s, 1459 s, 1437 m, 1383 m, 1303 w, 1287 m, 1248 s, 1178 s, 1160 m, 1097 m, 1056 m, 1031 s, 889 w, 831 s, 746 m, 698 m, 624 w, 605 w, 564 w, 532 m, 508 m, 461 m, 415 w
Anal. Calcd. for $C_{37}H_{43}Cl_2FeOPRu$ (762.1): C 58.28, H 5.68; found: C 58.23, H 5.70

Decomplexation Reaction

Since direct determination of the enantiomeric excess of **3a–c** was not successful, decomplexation reactions were performed to recover ligands **2a–c**. Therefore, triethylphosphine (0.1 mL, excess) was added to a solution of **3a–c** (20 µmol) in DCM (0.5 mL). The solution was stirred for 72 h at rt. Then solvent and excess of triethylphosphine were removed under reduced pressure. After redissolving in THF (1 mL), $BH_3{\cdot}SMe_2$ (2.0 mol·L^{-1} in THF, 0.1 mL) was added. After stirring for 20 min, water was added and the organic layer was separated and concentrated under reduced pressure. After column chromatography on silica with hexane/ethyl acetate 9:1, the phosphine boranes were analyzed by chiral HPLC to determine the enantiomeric excess.

Table S1. Decomplexation reactions of **3a–c** with initial and final enantiomeric excess.

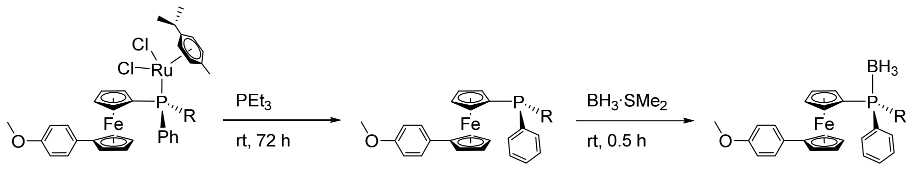

3a–c **2a–c** **1a–c**

R	initial ee / %[a]	final ee / %[b]
2-methoxyphenyl (**1a**)	97.8	97.5
2-biphenylyl[c] (**1b**)	98.9	98.0
n-butyl (**1c**)	95.2	94.3

[a] Before deprotection and complexation. [b] After decomplexation and reprotection.
[c] The *p*-cymene fragment is replaced by the incoming biphenyl group from the ligand.[4]

Deprotection-Reprotection Reaction Sequence

A solution of **1a–c** (30 µmol, > 95% ee in all cases) in diethylamine (1 mL) was heated in a J. Young valve NMR tube at 50 °C for 12 h. When the $^{31}P\{^1H\}$-NMR spectrum indicated full deprotection, the solvent was completely removed under reduced pressure. After redissolving in THF (1 mL), $BH_3{\cdot}SMe_2$ (2.0 mol·L^{-1} in THF, 0.1 mL) was added and complete reprotection was confirmed by $^{31}P\{^1H\}$-NMR spectroscopy. Water was added and the organic layer was separated and concentrated under reduced pressure. After column chromatography on silica with hexane/ethyl acetate 9:1, the phosphine boranes were analyzed by chiral HPLC to determine the enantiomeric excess.

Table S2. Deprotection-reprotection reaction sequence of **1a–c** with initial and final enantiomeric excess.

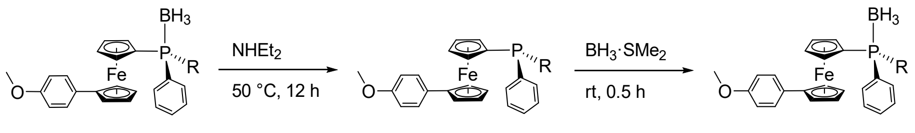

1a–c **2a–c** **1a–c**

ee > 95% ee > 95%

R	initial ee / %	final ee / %
2-methoxyphenyl (**1a**)	96.7	96.0
2-biphenylyl (**1b**)	98.9	98.9
n-butyl (**1c**)	95.6	95.0

Racemization of Ferrocenyl Phosphines

Thermal Racemization[5]

A solution of **2a–c** (0.2 mmol, > 95% ee in all cases) in toluene (16 mL) was heated at 110 °C for 15 minutes. After this equilibration, a 2 mL aliquot was transferred into a flask and cooled with ice, serving as the starting point of the racemization reaction (t = 0). Similarly, aliquots were then taken from the reaction at 0.5 h, 1 h, 1.5 h, 2 h, 3 h, 5 h and 7 h after the starting point. Each aliquot was cooled for 15 minutes before $BH_3{\cdot}SMe_2$ (2.0 mol·L^{-1} in THF, 0.1 mL) was added. After stirring for 20 min, water was added and the organic layer was separated and concentrated under reduced pressure. After column chromatography on silica with hexane/ethyl acetate 9:1, the partially racemized phosphine boranes were analyzed by chiral HPLC to determine the enantiomeric excess.
Following an approach by Mislow,[6,7] inversion is assumed to be a unimolecular process which obeys first order kinetics, ln(%ee) = kt. Therefore, the reaction rate of the racemization was obtained from the negative slope of the linear fit ln(%ee) versus t. Rearranging the Eyring equation, $k_{rac} = \left(\frac{[k_B T]}{h}\right) e^{\frac{-\Delta G^{\ddagger}_{110}}{RT}}$, to $\Delta G^{\ddagger}_{110} = (-RT)\ln[(hk_{rac})/(k_B T)]$, where k_B, h and R are Boltzmann, Planck's and ideal gas constants, yielded estimated inversion barriers for the ferrocenyl phosphines.

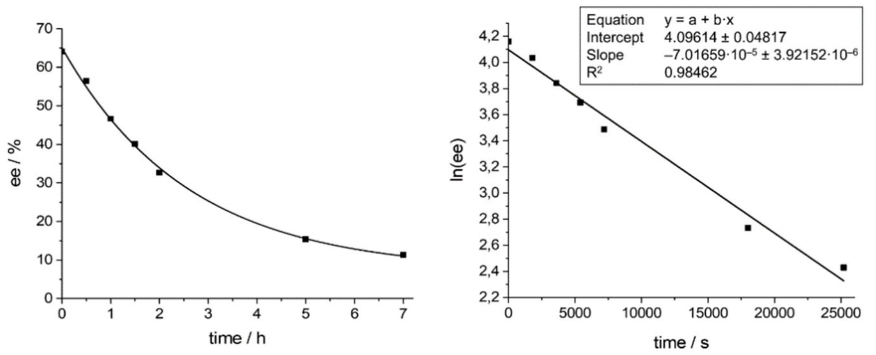

Figure S1. (Left) Decay of enantiomeric excess of **2a** over time in toluene at 110 °C.
(Right) Corresponding decline of the natural logarithm of the enantiomeric excess of **2a** over time.

Racemization by Single-Electron Oxidation[5]

Ferrocenium hexafluorophosphate (5 µmol) was added in one portion to a solution of **2a–c** (20 µmol, > 95% ee in all cases) in THF (1 mL). After stirring for 30 min at rt, $BH_3{\cdot}SMe_2$ (2.0 mol·L^{-1} in THF, 0.1 mL) was added, and after stirring for another 20 min, water was added and the organic layer was separated and concentrated under reduced pressure. After column chromatography on silica with hexane/ethyl acetate 9:1, the partially racemized phosphine boranes were analyzed by chiral HPLC to determine the enantiomeric excess.

Table S3. Racemization of **1a–c** by single-electron oxidation with initial and final enantiomeric excess.

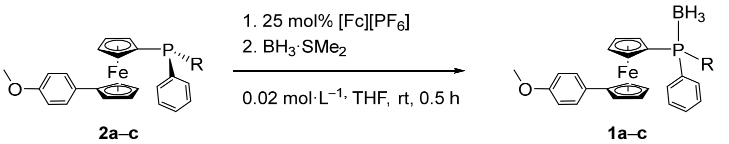

2a–c ee > 95% → **1a–c** ee < 10%

R	initial ee / %	final ee / %	reference ee / %[a]
2-methoxyphenyl (**1a**)	97.2	9.3	97.1
2-biphenylyl (**1b**)	98.9	0	98.9
n-butyl (**1c**)	95.0	4.6	94.7

[a] Reference experiment was performed simultaneously without adding ferrocenium hexafluorophosphate.

Racemization by Column Chromatography

A solution of **2a**, **2b** or **2c** (40 µmol, > 95% ee in all cases) in DCM (2 mL) was subjected to column chromatography on silica or neutral alumina, serving as the starting point of the racemization reaction (t = 0). Using degassed hexane/DCM 1:2 as solvent system, aliquots from the analyte band were taken from the column at 15, 25, 45 and 60 min after the starting point by opening the outlet valve of the column only at the specified times. $BH_3 \cdot SMe_2$ (2.0 mol·L^{-1} in THF, 0.2 mL) was added to each aliquot, and after stirring for 20 min, water was added and the organic layer was separated and concentrated under reduced pressure. After column chromatography on silica with hexane/ethyl acetate 9:1, the partially racemized phosphine boranes were analyzed by chiral HPLC to determine the enantiomeric excess.

Racemization by Acids and Trisilanol-isooctyl-substituted Polyhedral Silsesquioxane (PSS)

Acid (0.1 mL) was added to a solution of **2a** (20 µmol, 97.8% ee) in CD_2Cl_2 (0.5 mL). After 30 min at rt, water was added and the organic layer was separated by adding ethyl acetate. The solvent was completely removed, and after redissolving in THF, $BH_3 \cdot SMe_2$ (2.0 mol·L^{-1} in THF, 0.1 mL) was added. After stirring for 20 min, water was added and the organic layer was separated and concentrated under reduced pressure. After column chromatography on silica with hexane/ethyl acetate 9:1, the phosphine boranes were analyzed by chiral HPLC to determine the enantiomeric excess.

Table S4. Racemization of **2a** by acids and PSS with final enantiomeric excess.

acid	final ee / %
HBF_4 in Et_2O (50–55% w/w)	0.9
trifluoroacetic acid	0.3
HCl in Et_2O (2 mol·L^{-1})	90.9
H_3PO_4 in H_2O (85% w/w)	92.5
trifluoromethanesulfonic acid	–[a]
PSS-trisilanol-isooctyl substituted[b]	97.0

[a] Addition of trifluoromethanesulfonic acid led to irreversible oxidation.
[b] 1,3,5,7,9,11,14-Hepta-isooctyltricyclo[7.3.3.1^{5,11}]heptasiloxane-endo-3,7,14-triol (CAS 444619-08-3).

X-ray Crystallography

X-ray diffraction studies were performed with an Oxford Diffraction CCD Xcalibur-S diffractometer using Mo-K_α radiation (λ = 0.71073 Å) and ω-scan rotation. Data reduction was performed with CrysAlis Pro[8] including the program SCALE3 ABSPACK[9] for empirical absorption correction. Structures were solved by direct methods and refined by full-matrix least-squares techniques against F^2 with the SHELX program package.[10] All non-hydrogen atoms were refined with anisotropic thermal parameters and all hydrogen atoms were assigned riding isotropic displacement parameters and constrained to idealized geometries. Structure figures were generated with Mercury 4.0.0.[11,12] CCDC 1948526 (**1c**) and 1948527 (**3c**) contain the supplementary crystallographic data for this paper. These data are provided free of charge by the Cambridge Crystallographic Data Centre.

Table S5. X-ray crystallographic data of **1c** and **3c**.

	**1c**	**3c**·0.5Et_2O·0.5C_7H_8[a]
empirical formula	$C_{27}H_{32}BFeOP$	$C_{42.5}H_{52}Cl_2FeO_{1.5}PRu$
formula weight [g·mol^{-1}]	470.15	845.63
T [K]	130	130
crystal system	orthorhombic	monoclinic
space group	$P2_12_12_1$	$P2_1$
a [Å]	7.3512(4)	12.6314(1)
b [Å]	17.6832(9)	19.2701(2)
c [Å]	18.0660(9)	16.3939(2)
α [°]	90	90
β [°]	90	100.302(1)
γ [°]	90	90
V [Å^3]	2348.4(2)	3926.08(7)
Z	4	4
ρ_{calcd} [g·cm^{-3}]	1.330	1.431
θ_{max} [°]	28.291	32.587
F(000)	992	1752
reflns collected	9626	78422
independent reflns	4806	25991
*R*1/*wR*2 [I>2σ(I)]	0.0610/0.0780	0.0380/0.0745
*R*1/*wR*2 (all data)	0.1149/0.0909	0.0506/0.0797
Flack parameter *x*	0.05(2)	–0.030(6)
Largest diff. peak/hole [e·Å^{-3}]	0.595/–0.381	0.707/–0.607

[a] The solid-state structure of **3c** contains one diethyl ether molecule and one heavily disordered toluene molecule (isotropic refinement) per asymmetric unit.

Computational Details

All calculations were carried out in the gas phase using the ORCA program package (version 4.0.0.1).[13,14] Geometry optimizations and frequency analysis, to prove the absence of imaginary frequencies, of the tetrahedral phosphines were obtained employing the BP86[15–17] functional in conjunction with a def2-TZVP[18,19] basis set. For the inversion process of the phosphines relaxed surface scans at the same level of theory were utilized to find approximate transition states geometries. The presence of transition states was confirmed by frequency analysis. For the calculation of reliable energies, single point calculations, employing the PWPB95[20] functional and def2-QZVP[18,19] basis set, were used. The double-hybrid functional was chosen, since it showed excellent performance in large benchmark studies for a variety of main group and organic chemistry.[21,22] For the investigation of the reaction pathways, geometry optimizations and frequency analyses were carried out (BP86,[15–17] def2-TZVP[18,19]) and for the reaction energies frequency analyses were utilized. For all calculations atom-pairwise dispersion corrections with the Becke-Johnson damping scheme (D3BJ) were utilized.[23,24] Density fitting techniques, also called resolution-of-identity approximation (RI), were used for GGA calculations, whereas the RIJCOSX[25] approximation was used for double-hybrid calculations.

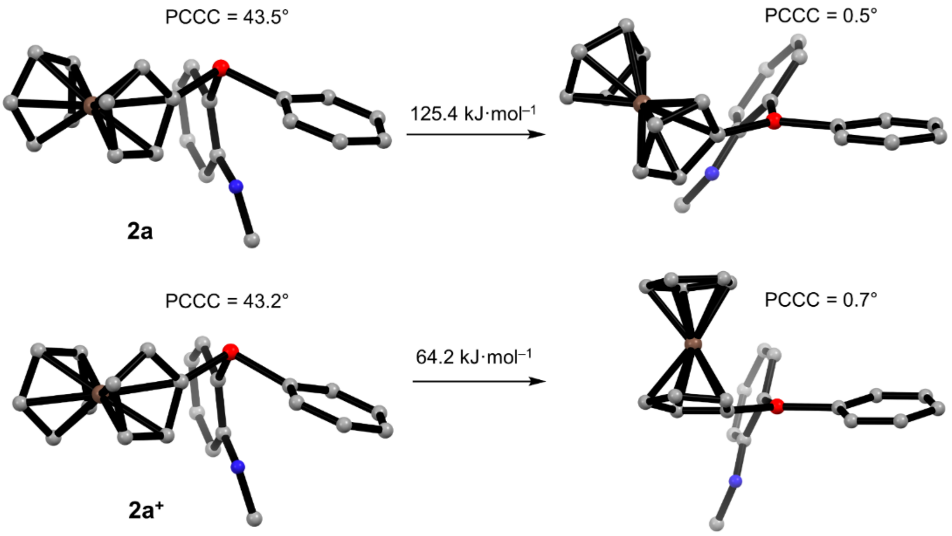

Figure S2. Geometrically optimized structures of **2a**. Given energy values are activation barriers for the pyramidal inversion. (Top) Tetrahedral and planar form for neutral ground state. (Bottom) Tetrahedral and planar form for oxidized transition state.

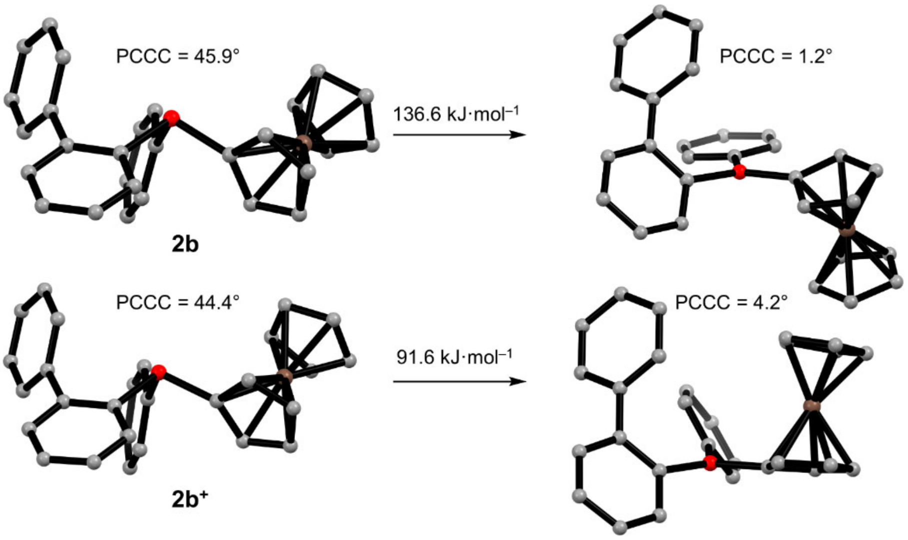

Figure S3. Geometrically optimized structures of **2b**. Given energy values are activation barriers for the pyramidal inversion. (Top) Tetrahedral and planar form for neutral ground state. (Bottom) Tetrahedral and planar form for oxidized transition state.

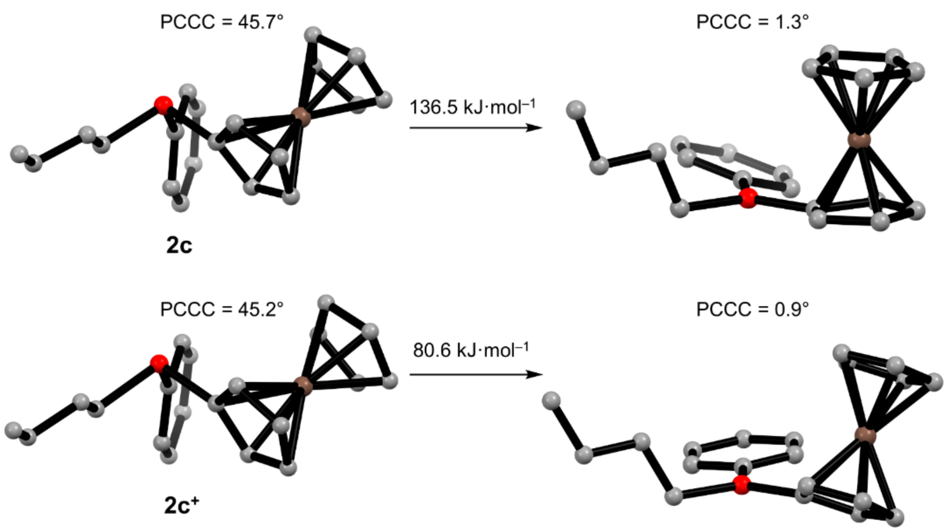

Figure S4. Geometrically optimized structures of **2c**. Given energy values are activation barriers for the pyramidal inversion. (Top) Tetrahedral and planar form for neutral ground state. (Bottom) Tetrahedral and planar form for oxidized transition state.

2a (tetrahedral)

E = –2914.881642990037 Eh

C	1.20560465034092	10.50222077384511	23.59230011630684
C	1.29116436818315	11.48335164891450	24.58860074689577
C	0.25972209408034	12.39625039048885	24.82396273420711
C	–0.88260589504647	12.34489353338516	24.02759144206515
C	–0.99020573666761	11.39980790949707	23.00281024221836
C	0.04495419846096	10.48078000915355	22.78174761456457
O	–0.00230541960546	9.53187771781831	21.80401982388108
C	–1.05671388977588	9.59245059891624	20.84689051088762
P	2.64917394177273	9.36452043181320	23.46049053662884
C	2.87000038890490	9.24632618801121	21.64014289271404
C	3.00109159624353	10.43010121674732	20.89489123627681
C	3.23216905683819	10.38308831624800	19.52060750500807
C	3.35845146689002	9.15107600172347	18.86979609532485
C	3.25116807824103	7.96978825889006	19.60775278486413
C	3.00743112056069	8.01551447825760	20.98271819358887
C	1.98584019152078	7.72349263611292	23.87502075593792
C	2.70162411267704	6.81730829431910	24.74462783709610
C	1.92079246534302	5.63007965898905	24.90589106896211
C	0.71806920291466	5.78739323668715	24.14390602799057
C	0.75300351397000	7.06965965576541	23.50915110856964
Fe	0.88074339886319	7.28043363071106	25.53602807907215
C	–0.92159273725954	7.44339559962799	26.47627821743755
C	0.02557527036835	6.77062057528105	27.33215934438641

C	1.12720844669047	7.68010293343798	27.52707644594739
C	0.85423560564427	8.88686516023184	26.81088789331885
C	–0.41254868467569	8.74218884936982	26.15875520232584
C	–0.08320991236127	5.40483190308429	27.85508272948361
C	–1.00918301438326	4.49132950859837	27.32915662464593
C	–1.11224011565259	3.18596820911092	27.81212759238039
C	–0.27181492911361	2.76162028949399	28.85133870154426
C	0.65867042173877	3.66106917316698	29.39360122530133
C	0.74520072213190	4.95733841813360	28.90259259954535
O	–0.28439159021816	1.50664348060927	29.39916602982303
C	–1.22570614013584	0.57351368770376	28.87460735844033
H	–1.07887278315261	–0.35119215648817	29.44323109940009
H	–1.04522443702963	0.37871645990861	27.80423356656826
H	–2.26120642771942	0.92769539470525	29.01022055786197
H	1.30077144954977	3.32241928460817	30.20718213018377
H	–1.84345973442623	2.51046772061151	27.37138459483998
H	–1.65789250740089	4.79850421023949	26.50813365042147
H	1.46402698151665	5.64497403160685	29.34926448308688
H	1.50954273188332	9.74864611598175	26.74110694510800
H	2.03392813535632	7.47057284243229	28.08676591549163
H	–0.88648980400859	9.48144103320878	25.51963093854632
H	–1.86226340684118	7.02971261466745	26.12469838052139
H	3.65952471623374	7.03029888287748	25.21107449409342
H	2.16405946847713	4.78301877460114	25.54146891216695
H	–0.09534294053218	5.06875341060810	24.08880448839513
H	–0.00973003908577	7.49986890861748	22.87183162536741
H	2.90362531464845	11.39654115429796	21.39494652033844
H	2.90501810192503	7.08773841483493	21.54719720218483
H	3.31823727238052	11.31209866835323	18.95405696839100
H	3.35061147263682	7.00348643278185	19.10979574847490
H	3.54343346630288	9.11386440333279	17.79531097583826
H	2.20182737360854	11.52134972896883	25.19015578226465
H	–1.88669176024634	11.37958049566411	22.38509220075722
H	0.35259712156694	13.13996097993297	25.61530611470569
H	–1.70111637353036	13.04731005071527	24.19188305834513
H	–2.03691153784934	9.39097212901786	21.31004785770484
H	–0.82944733459672	8.81212032879335	20.11290634691237
H	–1.08134976715039	10.57341931097650	20.34648312438932

2a (planar)

E = –2914.833865518156 Eh

P	1.65080820563919	8.96606491368711	22.68916238074643
C	3.07607837934699	8.92724760883173	21.63808354076974
C	0.99829845589338	10.54265949993604	23.19328897778091
C	0.96252809868388	7.41430600562293	23.20018399292100
C	1.86144765700781	11.53584093809914	23.69470244880926
C	1.38681208545794	12.79573336705191	24.05823142629935
C	0.02159891331150	13.07123668590164	23.96590338678219
C	–0.86287292861249	12.10011524709710	23.48416273309414
C	–0.38319811725081	10.84927566781896	23.08162585027247
O	–1.17604369837958	9.86412167989289	22.56541996982368
C	–2.55644639808838	10.15523157386028	22.36852987133078
C	3.63109109259816	10.10764786460683	21.09397058226092
C	4.75182051126615	10.04314080881439	20.26989469324464
C	5.34712304149488	8.81576287841400	19.95803640183963
C	4.79350903913513	7.64337231954380	20.48412603762191
C	3.67700479290666	7.68867565367367	21.31504750164978
C	1.66210542462471	6.47307488541068	24.05605194136814
C	0.82373003635123	5.33154549834462	24.24188464880528
C	–0.39912437325918	5.55535073178486	23.52747277848129
C	–0.31668128822186	6.82622357143919	22.87922316829080
Fe	–0.08483122814075	7.04319442245789	24.90540060093021
C	–1.69557971846174	7.00268830925029	26.15591509414384
C	–0.49411254942150	6.66196813164446	26.87874442807620
C	0.40236683647207	7.78525642023019	26.74613772051813
C	–0.24126945474601	8.79653861360938	25.96776926221440
C	–1.53652162918946	8.31156908115982	25.59916929323762
C	–0.21692777515067	5.40075067654888	27.57383599984304
C	–1.01147311357248	4.26246343215118	27.37019307150954
C	–0.74730386414463	3.05433618700586	28.01691767767538
C	0.34023387326747	2.96115238431137	28.89745050523377
C	1.14514041908695	4.08939085953480	29.11719276494934
C	0.86595549138482	5.28425621684073	28.46694570898436
O	0.69201642928207	1.82919523854600	29.58187945008126
C	–0.11206270425686	0.66862010055774	29.38523187991765
H	0.32946102863029	–0.11157360180868	30.01469420189855
H	–0.09692938377788	0.34228994569741	28.33193783610592
H	–1.15524203966746	0.84468706712335	29.69639522255511
H	1.98333437347662	4.00708437477691	29.80963333571706
H	–1.38907863555346	2.19599727263488	27.82594513349599

H	–1.84904558951683	4.31014942014581	26.67333642215974
H	1.49536792824484	6.15254492239388	28.66386063596034
H	0.19454388126885	9.74501349345788	25.66897094037164
H	1.41395577231171	7.84256763908422	27.13618479540295
H	–2.26299156268151	8.83520740820151	24.98644519513877
H	–2.57220784412582	6.37117416853203	26.04664435438007
H	2.64808561580826	6.63439835181395	24.48105756180620
H	1.04404967622592	4.47482196658821	24.87311973998236
H	–1.25234807060386	4.88268477991585	23.50502188486227
H	–1.07631679317529	7.29978476728826	22.26818680793666
H	3.17246641279569	11.07315723311422	21.30904007760459
H	3.25710492779038	6.76380367318724	21.71218032292077
H	5.16176006949977	10.96827541268466	19.86032857105680
H	5.23793808667576	6.67506648856495	20.24651960566444
H	6.22441213071772	8.77342918897603	19.31246980748596
H	2.91861189509357	11.29507988815523	23.81039475256502
H	–1.92381255399538	12.33284985964827	23.40952564789952
H	2.07839849776691	13.54668332227783	24.44070956319164
H	–0.36775495930576	14.04211238261843	24.27401557858861
H	–3.06403818416884	10.37211785996316	23.32324776691685
H	–2.99069955682683	9.25344762196320	21.92316922616416
H	–2.69366406522204	11.00737961932081	21.68317022266121

$2a^+$ (tetrahedral)

E = –2914.651992512785 Eh

C	1.24311389157055	10.51239637013370	23.62261885624201
C	1.34013519544203	11.53172910820574	24.58134577686265
C	0.26413770555031	12.37367589974905	24.87052858996286
C	–0.92946104269432	12.21120681901484	24.16804081813828
C	–1.05166174042883	11.21985949182179	23.18966432323042
C	0.02374742030525	10.36349418670058	22.91622104727928
O	–0.04030239776931	9.35903799993507	21.99847032565684
C	–1.13545639700429	9.35109245074137	21.07396243353370
P	2.75487418977417	9.50503917075599	23.37405470345410
C	2.84912692395810	9.31511527976479	21.56120705959028
C	2.65396382218678	10.44828011266120	20.75060907768628
C	2.81363240112077	10.36167689900647	19.36967565630391
C	3.18271585619093	9.14836544782379	18.77829909727068
C	3.39765967478368	8.02345225686986	19.57975570215726
C	3.23714739090475	8.10537405636272	20.96341778008985
C	2.27916156556040	7.80427090904583	23.89039467196939

C	3.05699102140117	7.06455873496886	24.84291375661542
C	2.43949125768728	5.79427178873610	25.04896992228636
C	1.27210027280682	5.73457155178052	24.21169028346346
C	1.17532896251344	6.96990485156732	23.50286170997190
Fe	1.10586475202609	7.28674245592836	25.56885852333637
C	–0.81939947974631	7.45414365546320	26.31037151724443
C	–0.01196941125142	6.79501233694630	27.31017967565506
C	1.07779988463274	7.68834237746015	27.60714868093467
C	0.90667050926239	8.88833207347238	26.85180084853914
C	–0.27141813232402	8.74729250714367	26.05410635532013
C	–0.16558596806190	5.42981930212336	27.78402119691605
C	–1.24751291296201	4.62938669708547	27.36130324760853
C	–1.40903323472049	3.32583397793011	27.80908892342980
C	–0.47129474288757	2.77931645791413	28.70817231781617
C	0.61915213132777	3.56544296804916	29.14267129773747
C	0.76299521301699	4.86060278782541	28.68880771778756
O	–0.52642325661414	1.53171462002446	29.20898837611383
C	–1.61909626540992	0.67925443231425	28.82387745428782
H	–1.45394904140049	–0.26249059553710	29.35472440382255
H	–1.61146575175263	0.50150492136595	27.73826360027849
H	–2.58082916592489	1.11641682046334	29.13022325453374
H	1.32911098208607	3.12682869479793	29.84337359068438
H	–2.26059602317590	2.74108791600212	27.46755362831107
H	–1.98659746850033	5.03803588093793	26.67256126079126
H	1.60772390424586	5.45041492548179	29.04351157127392
H	1.57214341397299	9.74560942371698	26.85874005703279
H	1.89881375698264	7.48941071599499	28.28837524278258
H	–0.66020893747834	9.47952494995663	25.35300674464697
H	–1.70245328171039	7.04363231841372	25.83139641227923
H	3.94724913694079	7.43887047461803	25.34108005688253
H	2.77741777696512	5.02132406028790	25.73360175722073
H	0.57607549376855	4.90239344337251	24.14885591601892
H	0.39722065461900	7.26991599715475	22.81040355119663
H	2.36467275568077	11.39803289056507	21.20459855692149
H	3.40854944366653	7.21964013304907	21.57704276142635
H	2.65273419008197	11.24490819069464	18.75033730601485
H	3.69329265468378	7.07720227403245	19.12498650076456
H	3.30935054520150	9.08241502791646	17.69743697136624
H	2.29316817440518	11.66637233539062	25.09721562523761
H	–1.99043943735857	11.11470208211630	22.64836547913970
H	0.36461457450218	13.15747051615158	25.62048157585275

H	–1.77879647361321	12.86462877974205	24.37031831155577
H	–2.08693787810177	9.12204230689800	21.57913393617375
H	–0.90215817893780	8.56301499511377	20.35096037228771
H	–1.21431987999652	10.31672248597642	20.55334483101142

2a+ (planar)

E = –2914.627533831735 Eh

P	1.18310278921390	8.98909404562253	22.73380551350609
C	2.83706370445078	8.99693947686381	22.13146027341999
C	0.30781085444128	10.52456682882052	22.90348126492725
C	0.35365100085842	7.53491909014407	23.20901209428554
C	0.68930916761880	11.46657191593942	23.87480800624919
C	0.01359804880464	12.67873505214735	23.98624504237765
C	–1.05978447189227	12.94417470763850	23.13056702739866
C	–1.46145685513696	12.02151207763230	22.16312188643111
C	–0.77838301738392	10.80443241246119	22.03538043233602
O	–1.07696032230549	9.84124781090625	21.13133193520074
C	–2.13976907302233	10.08721253144555	20.19441422825540
C	3.50343942706881	10.23599907241168	21.99173126378644
C	4.81037240566939	10.26459842504586	21.51717165908626
C	5.47078875590251	9.07706575299570	21.18164815828184
C	4.80736345267855	7.85147523942843	21.30738058193920
C	3.49740494002662	7.80060144187893	21.77340709970225
C	0.91929162903409	6.25700377077280	23.59682273534243
C	–0.11645104974917	5.50653983825785	24.23232208655764
C	–1.29587851912537	6.31186021716670	24.28451425969684
C	–1.01684531759258	7.57223338861578	23.67393208032444
Fe	0.25577742337132	7.25545631831672	25.24844413948835
C	–0.47297537271013	7.46667959132147	27.14887687550476
C	0.63150216668799	6.53940394083413	27.16729442476730
C	1.77586795695106	7.25491706309204	26.65572669266206
C	1.38562919017502	8.59583004273515	26.37623823228548
C	–0.01034759193660	8.73069904355987	26.65852648891855
C	0.59025994902747	5.13932142916728	27.58671360084384
C	–0.47326288813210	4.64733668674464	28.35830574583697
C	–0.52953770744016	3.31362267910212	28.76121892041253
C	0.49811350715600	2.43177367071548	28.39045292830317
C	1.57451260900211	2.91047480777390	27.61847772474844
C	1.61558395211897	4.23846290561902	27.22917212579953
O	0.54889659414600	1.11847658247494	28.72396097941975
C	–0.51598429685491	0.58640485989354	29.52124888197774

H	–0.27295016559401	–0.47003279863397	29.67055239758887
H	–1.48237719220914	0.67344738061364	29.00025565943919
H	–0.57159713961458	1.09437441867909	30.49663327420629
H	2.36690871640447	2.21483746713873	27.34248383409282
H	–1.36619927998015	2.97374614617680	29.36829713877396
H	–1.26965302493274	5.32174176892629	28.67497366475551
H	2.45503471880694	4.58398102088256	26.62432018900828
H	2.03567824514476	9.36621916580105	25.97094894996043
H	2.77450693781763	6.84887884372773	26.52937963442426
H	–0.60851080955250	9.62778146764243	26.53098762799849
H	–1.49528185594219	7.24080036930702	27.43535268894107
H	1.93918925882031	5.92671570561237	23.43772327021679
H	–0.00118555588782	4.51494910567337	24.66075413086659
H	–2.24083205785203	6.02699585784791	24.73663985316348
H	–1.70116656532939	8.40584736322559	23.56005211207381
H	2.99234877795221	11.16449409935685	22.24643964945945
H	2.97879486289371	6.84514148385911	21.82657710365955
H	5.31878414056956	11.22283077613206	21.40855451428356
H	5.31072006394834	6.92613414996199	21.02630803473176
H	6.49578209043075	9.10758581954794	20.81282902399861
H	1.52232886058338	11.23934059160196	24.54013329631682
H	–2.29809357249141	12.25571997348037	21.50771296414647
H	0.31597152885121	13.40702252808087	24.73751796803960
H	–1.59902641383270	13.88824698936445	23.21454165097245
H	–3.09933736422696	10.21851885029199	20.71617669215373
H	–2.18105266992964	9.19573848438068	19.56201994991785
H	–1.91990657596985	10.97154225377551	19.57861033673709

2b (tetrahedral)

E = –3031.391527374495 Eh

C	5.92937842408816	15.97082574449240	4.50836323983670
C	5.44330228184551	15.87946882644264	3.19724824418028
C	5.99531467547804	16.74683103868038	2.23290538815455
C	6.98719846272434	17.65589200699415	2.56580263891215
C	7.46688841594472	17.73049171683894	3.88385434111559
C	6.93095658161504	16.88073016980508	4.85978138321651
C	4.40569902452187	14.90886245547436	2.83569823410606
C	3.59165315268578	14.92251190035965	1.64674613225815
C	2.73680134282226	13.77469460329524	1.66165191543062
C	3.01291355087805	13.03536721945270	2.85745953393553
C	4.03896819075288	13.72815780401874	3.57649659029770

Fe	4.70260624262568	13.20895996605758	1.71404904213306
C	6.58297443184430	13.34932056052219	0.94125144128436
C	5.63650751724768	12.98353594778441	–0.08426744408978
C	5.01988153901569	11.74483149581533	0.33141778420084
C	5.58290223942383	11.35749308685194	1.58796965778057
C	6.54552434817700	12.35152016217101	1.96554768793603
P	5.14519434639735	13.80817284255225	–1.62586442035584
O	8.44955330827946	18.65763575811445	4.11254326354298
C	8.95844104619514	18.75517110702399	5.44000180590360
C	5.69606451708229	15.54709456516807	–1.35104445189104
C	7.04321754366366	15.90542367777663	–1.18983111122117
C	7.40790504945846	17.24455860252809	–1.05132162928883
C	6.43116804931075	18.24488778930688	–1.08129036314940
C	5.08861978337103	17.89932902483490	–1.25010584648827
C	4.72547000075281	16.55741347128082	–1.38610398392249
C	6.50835080054612	13.26026825588816	–2.75558911359180
C	6.70167692311027	13.93050824806892	–3.98874387095495
C	7.74759020395940	13.52977533681375	–4.83081297060416
C	8.57810885617541	12.45987698154592	–4.49081500205997
C	8.37106100234852	11.78264162123295	–3.28832385631001
C	7.34811156279713	12.18652128187066	–2.42706942040579
C	5.84001505833314	15.07710021906081	–4.37808612406485
C	4.45633848051495	14.91299258222475	–4.54918874440920
C	3.64501330326362	16.00718294622665	–4.85325094627870
C	4.20390501409898	17.28058625467291	–4.98799921925631
C	5.58241098007542	17.45094295043204	–4.83604868556180
C	6.39416342271370	16.35606829935748	–4.54006879478173
H	4.24191781137475	11.22075928208422	–0.21714377308241
H	5.30267994649890	10.48488250385751	2.17128701999343
H	7.11802914084490	12.36884173487343	2.88897864353197
H	7.17480785476188	14.25915720862491	0.96193006862229
H	3.63530456765149	15.67225937166606	0.86343813160295
H	2.02762386418071	13.50078427654215	0.88601080527643
H	2.55027238904453	12.09763128021123	3.15188825967037
H	4.49664553772531	13.39328294556393	4.50273252379887
H	7.81182562712874	15.13141301806244	–1.20395306235945
H	8.45861960037896	17.51017036302469	–0.92427645675174
H	6.71755940092783	19.29266428670819	–0.98023837454179
H	4.32359485832391	18.67575901359038	–1.28907537141334
H	3.68169435230057	16.28628012323487	–1.55414388799673
H	7.89415635884237	14.06139702383582	–5.77299038375015

H	9.38052986058756	12.15679358707254	–5.16477674589316
H	9.01390926844072	10.94605404231202	–3.00983803662226
H	7.21246976734614	11.67465843366068	–1.47365857241506
H	4.01850937520575	13.92104537855734	–4.43629341283590
H	2.57113107890777	15.86431065332863	–4.98281933495266
H	3.56731770889062	18.13762338061014	–5.21220766842372
H	6.02584147969459	18.44270568701653	–4.93375614761183
H	7.46560854268122	16.49246225454961	–4.38834043343888
H	5.65675165541658	16.69198717384686	1.19886274985475
H	7.41247103542521	18.31728621715299	1.81119368294052
H	7.27682037708148	16.92055028203744	5.89127887324311
H	5.51044179398836	15.32811268863305	5.28381595708600
H	8.16818374679699	19.04161102679713	6.15397577647901
H	9.72444460128026	19.53785440377160	5.41083327555263
H	9.41552572413337	17.80601283773582	5.76647656889757

2b (planar)

E = –3031.339498475415 Eh

P	5.53968618374162	14.70213685996674	–0.72151085421410
C	4.59145014714007	13.44301205799490	0.06351046679707
C	4.80995556664339	16.20008599531556	–1.28749472784223
C	7.28041061522558	14.42432515383212	–0.99603525811205
C	7.00056706158407	15.19287217329243	3.47685440718039
C	5.86379766949133	15.62880595092347	2.77788412159914
C	6.04953736654367	16.62676339382940	1.79688292306855
C	7.30658183532402	17.14837515739416	1.52346578501886
C	8.43195993709879	16.68304742676598	2.21793972733475
C	8.27229836944354	15.70786155647367	3.21309266995756
C	4.54304958866513	15.05228226790984	3.02927307921084
C	3.29524290424597	15.47797912880344	2.44805667461802
C	2.25115386087436	14.61985393399650	2.91935081983999
C	2.83869203609789	13.64461254946441	3.78634685431532
C	4.24538327357147	13.90081096433178	3.84645966220980
Fe	3.67903313989252	13.53252728292890	1.91750337531703
C	5.13353460201579	12.46408487967533	0.98108408271939
C	3.17429641447286	13.17609833266104	–0.04373866805916
C	2.86486904407144	12.04073854735874	0.76906207280310
C	4.07301729515046	11.59427057650281	1.39133235918022
O	9.63163447366444	17.23289872776949	1.85379819665613
C	10.79805165128080	16.75005774888326	2.51244509139346
C	5.64562414554105	17.22656572839300	–1.78786784848773

C	5.10279689305882	18.43778000190380	–2.20285552953422
C	3.72684890416910	18.67734719435231	–2.11328546760104
C	2.89897265054351	17.67857589721839	–1.59177206328299
C	3.42228600772858	16.45220457449030	–1.18709463548786
C	7.87673131297851	14.56691781290287	–2.27917537391930
C	9.26741121127100	14.41150089935003	–2.38950964487929
C	10.05521739959996	14.07106659659671	–1.28937515845152
C	9.45936121172017	13.90286024724888	–0.03685870976276
C	8.09032589244940	14.10412118075204	0.11179360503026
C	7.08512400797802	14.87725244702534	–3.49372066417360
C	5.96615324342167	14.10498086446987	–3.84798879920931
C	5.21779690495207	14.42294614382930	–4.98022838040033
C	5.57389426733021	15.51540829715603	–5.77835735810560
C	6.69576080574062	16.27628672257141	–5.44787811510380
C	7.44556855459249	15.95742646065623	–4.31370040917298
H	2.46800256279941	13.72298156742083	–0.65816661618576
H	1.87760089006027	11.60560086865679	0.89730252768726
H	4.17073746860295	10.76198489299435	2.08242058448337
H	6.16909203546462	12.40459980659591	1.29850855936353
H	3.16823458486479	16.30224838611768	1.75267408947490
H	1.20177937246318	14.68626690907287	2.64632686967747
H	2.31582261769284	12.83601190231141	4.28922193079497
H	4.96759146934659	13.30894538535606	4.40087189061442
H	6.72205936056812	17.07156635259223	–1.83508848203596
H	5.76810485771741	19.20958743503745	–2.59334046397304
H	3.30838561537422	19.63022601230663	–2.43714045924258
H	1.82489663801313	17.85058847417085	–1.50224040271108
H	2.75697355393821	15.69715558754926	–0.77472377413920
H	9.72461048695787	14.52014542194942	–3.37431664295493
H	11.13075891811563	13.93745692618985	–1.41195686954522
H	10.06388573511320	13.64959855684767	0.83515443160264
H	7.64275794896351	14.06547140768218	1.10388748589060
H	5.68968732897795	13.25017676747056	–3.23101153764655
H	4.35338843147191	13.81219275212843	–5.24423837041925
H	4.97910074885798	15.76820955311633	–6.65705297216294
H	6.98195705549383	17.12912594410930	–6.06484292346138
H	8.30776634332381	16.56798180116367	–4.03900623733597
H	5.19563099855543	17.01047872463479	1.24175967967466
H	7.43794475737270	17.90810743809120	0.75298442086360
H	9.12474241718503	15.34000632659970	3.78146106419801
H	6.89676832588768	14.43375661266868	4.25300319615258

H	10.76331043788731	16.95158944149636	3.59617031466985
H	11.64011035719217	17.29156060955355	2.06782123453207
H	10.93075923242386	15.66763340112564	2.34802216368393

2b+ (tetrahedral)

E = –3031.165528312395 Eh

C	5.78407658388628	15.99734183856782	4.59458098941659
C	5.33579237217566	15.94513754249406	3.25976606358237
C	5.91184874124684	16.84058254205315	2.32589630565239
C	6.88706394465499	17.73731802951579	2.70949668960304
C	7.32098033470050	17.78263922423710	4.05289451064321
C	6.75743222245581	16.90222835083182	4.99805084332806
C	4.32049439925468	14.99104259795869	2.84547142573073
C	3.63468907059415	14.98240807997359	1.57978168831508
C	2.82480873462679	13.80658833418088	1.50487140463575
C	3.02025062048624	13.05942025630263	2.70911860011566
C	3.95358306540119	13.77241928091389	3.52414925702149
Fe	4.80550253762408	13.28457752923719	1.70021911244916
C	6.71468095470281	13.42577444878549	0.92404580126363
C	5.82923695628657	12.98244841252125	–0.11440284856221
C	5.22605092551581	11.75814093827434	0.34442393152619
C	5.75384591375842	11.44131625099043	1.63358918511796
C	6.67133064986710	12.47851100010326	1.99992509210573
P	5.30548485923542	13.83659907575876	–1.63228889510014
O	8.27149888669390	18.69708708289259	4.32912413657591
C	8.74819602819678	18.80833695319570	5.68140110892477
C	5.91223906278561	15.54762639622166	–1.37110007260246
C	7.27560560980588	15.86022864789175	–1.25425380351894
C	7.68014255141243	17.18644319748350	–1.09869786961865
C	6.72937300649628	18.21252382841017	–1.07554435273400
C	5.37217168826768	17.91008897406094	–1.21101333929598
C	4.96569768764308	16.58189151509746	–1.35879660728592
C	6.51085226343888	13.18509149809781	–2.85299627278987
C	6.60609833052059	13.84921646562170	–4.10253584189936
C	7.56777056925767	13.41738233105841	–5.02525715435405
C	8.38709719472974	12.32156212812070	–4.74977815696808
C	8.26473795993223	11.64802571576596	–3.53184895471972
C	7.33479832529556	12.08168348333093	–2.58636698545462
C	5.73507899654213	15.00924292184185	–4.40872102259792
C	4.33534107407604	14.87270995073261	–4.41561145061411
C	3.51758616737684	15.98319559640667	–4.63368113135342

C	4.08653474789687	17.24099759011158	–4.84448475004838
C	5.47842048640639	17.38172761137010	–4.85893469333014
C	6.29690882745532	16.27267546826626	–4.65131435253185
H	4.46709136325929	11.19726817498966	–0.19468137857144
H	5.48190877536775	10.58289722494344	2.24096085736298
H	7.23097845462120	12.54240482962397	2.92881627812593
H	7.27922640725374	14.35294906743997	0.90980649519631
H	3.72587551543889	15.74057627630047	0.80910901035886
H	2.19624216473872	13.52490599363117	0.66516012686518
H	2.56771697640781	12.10233697617149	2.95118153066619
H	4.33725462767388	13.43807506399316	4.48306764383790
H	8.02210395873313	15.06694063237254	–1.32068119004345
H	8.74149684762895	17.42282706388347	–1.01244559293406
H	7.04902626952463	19.25011437635592	–0.97210274321233
H	4.63036193831500	18.70913879532291	–1.22059600981219
H	3.91131251790907	16.34568836720320	–1.51231770136671
H	7.64822870251153	13.93740224384642	–5.98092616260640
H	9.11609797840162	11.98920005325847	–5.48924175707483
H	8.90413561651765	10.79313232972755	–3.30981761852001
H	7.27034831273269	11.57101427074534	–1.62525938683496
H	3.89210264163019	13.88691879306601	–4.27182019079909
H	2.43380483230698	15.86235325871803	–4.65075803477013
H	3.44816126098098	18.10913592844175	–5.01163686554680
H	5.92674646374425	18.36128341178368	–5.02765491984688
H	7.38174415706434	16.38498090412666	–4.63877838432858
H	5.60209137615058	16.81601769339895	1.28120753310854
H	7.33666018129333	18.42035865892856	1.98967058167817
H	7.06899008933917	16.92783514162437	6.04018328129868
H	5.34372550264321	15.33355685506244	5.33900630339525
H	7.92873142809581	19.08235153703248	6.36216966021446
H	9.49596021733877	19.60623935611993	5.66163164584516
H	9.21511007167358	17.86786263320946	6.00972239768636

$2b^+$ (planar)

E = –3031.130657768183 Eh

P	7.14906179761144	14.05762382489289	–1.47517260124509
C	6.91697617356518	13.10295288938531	–0.03438217108132
C	7.46815102804229	15.80269004753256	–1.23601159612423
C	6.85544163508154	13.20342789165754	–3.01824748475126
C	5.31626568746623	16.11210060929316	4.18222940428370
C	5.24016005298230	16.02253875044840	2.78413758530294

C	5.93716362437138	16.98375514720806	2.02328933581704
C	6.67556008379495	17.98225729885243	2.63427571143013
C	6.74466882499438	18.05819476988595	4.03840869207264
C	6.05553131583156	17.11237958057455	4.81318007148328
C	4.46242723672332	14.96497897330443	2.13906010941224
C	4.05412670292797	14.90702569461484	0.76364207979442
C	3.34373725974831	13.68495610684582	0.53526840145939
C	3.30353568822450	12.96755778594269	1.77385425469200
C	3.99818452046050	13.74455853364667	2.75194820103550
Fe	5.25028336496900	13.26583765312380	1.18846346663961
C	7.27400116310045	13.47521474549420	1.31633977350072
C	6.19160664710320	11.84913286742219	0.02773493026731
C	6.17246802016791	11.43075860470507	1.39185761934623
C	6.83356183193790	12.42643365816007	2.18032680443040
O	7.49446573201523	19.07265974835711	4.53901314779151
C	7.58123018052668	19.20183905628559	5.96355769344117
C	8.62983965030303	16.12348090089409	–0.50213954072050
C	8.93303681306590	17.45532130212010	–0.22874958836164
C	8.10517951013713	18.47557213324543	–0.70537103134101
C	6.96194432266398	18.15967053697263	–1.44392462615057
C	6.63161377681854	16.83013080566203	–1.70549052313546
C	5.99500983316124	13.66046398799307	–4.04423445703224
C	5.99178936664558	12.96501750110380	–5.26299717020408
C	6.76719487567118	11.82140945058028	–5.44848059171659
C	7.57561098637166	11.34508295341293	–4.41132992054812
C	7.63624113992281	12.04143533822895	–3.20903869556052
C	5.06150156260716	14.78822358682705	–3.84671255174866
C	4.14322969638753	14.74987468432603	–2.78373405501139
C	3.23281597736761	15.79129021274058	–2.59736669037144
C	3.23928367038356	16.88910974985730	–3.46305242255639
C	4.14573794461059	16.93095742513476	–4.52721913324589
C	5.04515745027859	15.88226903485058	–4.72570621497121
H	5.76776721296671	11.32200671274363	–0.82096696248100
H	5.70264194911939	10.52714099364806	1.76815002172777
H	6.95456403193027	12.41219425314074	3.25924337600276
H	7.77413426476202	14.39023628493418	1.61737343720520
H	4.27916266258665	15.65614350889104	0.01026050286200
H	2.89074363201806	13.36239112609702	–0.39707403340026
H	2.84465314332191	11.99713422331076	1.93735004894558
H	4.17551170954059	13.44666242833299	3.78061243506848
H	9.29975016033442	15.33195258356455	–0.16534848111686

H	9.82929285336634	17.69585873250360	0.34304220316626
H	8.35447711595109	19.51789030329665	–0.50465687477387
H	6.31069872927076	18.95191159883419	–1.81372673620143
H	5.72726353078321	16.59827574743285	–2.25909963010670
H	5.32909647580088	13.31042903730540	–6.05733746416968
H	6.73190103150904	11.29259526326180	–6.40116003974365
H	8.18194413660276	10.44993829580890	–4.54877802683005
H	8.31144322194861	11.70945814052266	–2.41954694353482
H	4.13561545884759	13.88179575300944	–2.12394628559651
H	2.50803240485673	15.74316094949323	–1.78403157026389
H	2.53083078103796	17.70452008155760	–3.31601042808279
H	4.15179999256411	17.78433639649100	–5.20577528556701
H	5.76004300080038	15.92013609012759	–5.54864058938595
H	5.91181281488619	16.94075403727361	0.93419298925588
H	7.21336079235642	18.72382920153329	2.04499355733786
H	6.08007760708252	17.15352640303970	5.90025156319960
H	4.76914656751721	15.40034647589236	4.80133229034809
H	6.58792149490281	19.37463281911367	6.40609558407947
H	8.21954368638649	20.07243201090678	6.14281302060333
H	8.03951138890650	18.30812370634937	6.41531510512942

2c (tetrahedral)

E = –2726.565206896687 Eh

C	2.10112592555177	16.19905920452781	0.08436032475517
C	2.16638321384110	17.61086138430259	0.31343529968522
C	2.70348351887547	18.23008516876506	–0.86361427120978
C	2.97530460495330	17.19945015980960	–1.81799935139440
C	2.60088841253236	15.92915474310286	–1.24206543858554
Fe	4.02050742775690	16.85498689178011	–0.10353104515863
C	5.97039668617335	16.47897843633658	–0.58812783698049
C	5.84987376309792	17.75878796023443	0.04509416920415
C	5.28116946819419	17.55627994101961	1.34313754516581
C	5.04843611489903	16.14642685515097	1.53039515967771
C	5.47682991693165	15.48920739620984	0.32099041907184
C	4.44646338129457	15.51548522144153	2.70907846031027
C	3.87979885788994	14.22728477279548	2.63828782610432
C	3.30098170358193	13.63350615940190	3.74906583692284
C	3.26609347473651	14.31132279304678	4.97864304853322
C	3.82567598886978	15.59153415720573	5.07372597493453
C	4.40860357933415	16.17515281088347	3.94502426108351
O	2.66965019304240	13.64227160055331	6.01531871502979

C	2.61874398882741	14.30448985092021	7.27538231753975
P	2.78907837871887	14.37737853871823	–2.17689092856509
C	1.04596882768217	14.20557065201058	–2.83190359836581
C	0.74476581171319	15.24130094774791	–3.91730690221635
C	–0.66106112215726	15.09715037803640	–4.50860318465717
C	–0.96342949150347	16.14323743551727	–5.58276428921883
C	2.77796876259332	13.10295459031088	–0.84191394555937
C	3.93193367340365	12.31542342002412	–0.69864529016640
C	4.00572735212262	11.31604090489540	0.27512085053484
C	2.91482909605036	11.07942190489806	1.11471434348495
C	1.75535949458411	11.84769225963772	0.97661850492624
C	1.68870613623795	12.85029977233927	0.00762621675923
H	3.63004830037511	14.52043398537338	7.65849901799284
H	2.11087839996656	13.61430513413665	7.95779792945130
H	2.04701932675257	15.24558435368724	7.21349425098538
H	2.85976974494764	12.63937715638639	3.68315786031351
H	3.87466398601946	13.69064154855714	1.69039364727821
H	3.82121815836572	16.13886876872167	6.01464489018509
H	4.85701720661419	17.16486122988977	4.03929686872874
H	5.02533507933998	18.34028426975145	2.04955600128123
H	6.11277950878420	18.71721548342512	–0.39311710492632
H	5.41989035447005	14.42258482092121	0.12990340656020
H	6.34165204711331	16.29091730660020	–1.59117021799202
H	3.41656173022139	17.33143462032527	–2.80196497142227
H	1.89419063018403	18.11734106164594	1.23532849376464
H	2.90324983515052	19.28991262536340	–0.99357911840717
H	2.96752712370379	10.29816433251623	1.87396566331432
H	4.91360083236438	10.71981908029774	0.37543436796174
H	4.77707283188132	12.49026047763904	–1.36786098699822
H	0.90082191485039	11.66924453662507	1.63100524563453
H	0.77573134354976	13.43900730259751	–0.08630085709151
H	1.79300006368151	15.45319323568638	0.81075344989378
H	–0.88634875176268	17.16237974956311	–5.17467708261523
H	–1.97653678709668	16.02251004869701	–5.99206769957006
H	–0.25318191365783	16.06821056846226	–6.42012290587152
H	–0.77496252742072	14.08436695462897	–4.93081638368631
H	–1.40301843891062	15.17318092388465	–3.69583351677606
H	1.49346878391337	15.15946818442021	–4.72387808436691
H	0.85849708706625	16.25417996674789	–3.49650416055005
H	0.96387061344887	13.18454996606688	–3.23931656820029
H	0.31260737625490	14.29330199575848	–2.01491662651715

2c (planar)

E = –2726.513217361449 Eh

P	1.21548006855873	14.93114107934399	–1.12337827709844
C	1.42572258319892	16.46221515667787	–0.27826932462674
C	0.49048142917345	14.95287013351883	–2.79592486627971
C	1.62920751992762	13.38724807262587	–0.38282551167592
C	1.39400297551167	16.74328466608590	1.13711765042149
C	1.53970089960931	18.15606208505128	1.31979860583603
C	1.67099244375381	18.76450944123560	0.02923840252374
C	1.62993447839050	17.72535090721875	–0.95631094404869
Fe	3.16157846387633	17.40327376907971	0.33586792493886
C	4.84010801558436	17.22014266798456	–0.81519925830957
C	4.93311605109597	18.37686267684389	0.02990438336604
C	4.83153903771508	17.93960577649038	1.38869301431989
C	4.68296265153183	16.50556506052220	1.39558143660323
C	4.69075804328114	16.07043598477123	0.02255993682850
C	4.56477159850739	15.64783735974102	2.58036803184965
C	5.11214581797531	14.35167124506575	2.58464351848100
C	5.01085350466075	13.52907080018594	3.69983278758730
C	4.34976914220469	13.98068955252020	4.85146812091527
C	3.80040060020131	15.27162600738586	4.87027460845560
C	3.91530057112046	16.08640769881131	3.74312686340943
O	4.28882519496937	13.09535375160191	5.89421468774440
C	3.61674210638706	13.52154418896656	7.07687254853755
C	1.48082139042653	14.76543476390836	–3.95434952535642
C	0.79160405886839	14.75292448101243	–5.32157064315737
C	1.77376363934791	14.56441467965953	–6.47912736545181
C	1.33219304063701	12.17831395508186	–1.05842856250335
C	1.65002696047192	10.95188866067532	–0.48249597672950
C	2.26569620234748	10.88622278901427	0.77309117553049
C	2.56764761502975	12.07733559186530	1.44158297127469
C	2.26242672597869	13.31453363965319	0.88028778079197
H	4.10483741179488	14.40503372928838	7.52103528211014
H	3.67908648214441	12.68059068949463	7.77603745816598
H	2.55764153320290	13.75370809179563	6.87587295850413
H	5.44041753258173	12.52691366720481	3.70146525706008
H	5.62922756178168	13.98821620189205	1.69602776686561
H	3.27698764214533	15.64514512699622	5.74868910997111
H	3.46343640159487	17.07946170185936	3.75580621703945
H	4.86694695589319	18.57232630120238	2.27100019272426
H	5.04105384573765	19.40526103527946	–0.30324278023963

H	4.53574945111183	15.04973493779245	–0.31760229517476
H	4.85356720839356	17.21875437471270	–1.90136404120967
H	1.73475635797853	17.85468405135861	–2.02955982215397
H	1.55747022135725	18.67334304578509	2.27521116140792
H	1.81127810522786	19.82290214145553	–0.17038624243675
H	2.50828840383537	9.92250671098041	1.22092412971515
H	1.41158868490257	10.03415260769806	–1.02328468737407
H	0.85062786583753	12.20200428469309	–2.03721590443160
H	3.05535564328164	12.05384676111915	2.41682128596769
H	2.52867824731678	14.22439066308448	1.41617965631233
H	1.28119586969841	16.00669644719685	1.92651585649105
H	2.51923310383827	15.37363210273515	–6.50189881801522
H	1.25769140221534	14.55749522452953	–7.44964425978722
H	2.32030000025457	13.61414169305653	–6.38323454133647
H	0.03685551622646	13.94815064015286	–5.33944004518637
H	0.23351206301833	15.69546945042769	–5.45575458442045
H	2.03673181981196	13.82501869482496	–3.80827716648638
H	2.23344480485601	15.56955674769435	–3.91622747159355
H	–0.30140352315985	14.18666936896933	–2.83556778060842
H	–0.02644844322184	15.92275286412148	–2.87177208605807

2c⁺ (tetrahedral)

E = –2726.339653586108 Eh

C	1.96652709090362	16.13617435520379	0.17265159155408
C	2.02858654665459	17.53659499784201	0.47678447427725
C	2.50543131938321	18.21480041213552	–0.69210316563114
C	2.74503838105362	17.23023100563713	–1.69942437722246
C	2.40159215195115	15.93304604796699	–1.17826612660583
Fe	3.90749182794772	16.84856883979727	–0.00390254991014
C	5.83050870760235	16.48146094102010	–0.63834193828596
C	5.75753960045034	17.76231956035436	–0.00388995265297
C	5.31766557165312	17.56432707404358	1.34144144334967
C	5.14883845922747	16.14921821616360	1.56601606373422
C	5.43744759756989	15.49352296135655	0.31636523360520
C	4.58391248936679	15.52476935179621	2.74841098058163
C	4.05549731007088	14.21089588331011	2.69423513140530
C	3.50683715551501	13.62075315351137	3.81263485935839
C	3.47365070886034	14.31946414853503	5.04069249555732
C	3.99741099386148	15.62691848518976	5.11606931021037
C	4.53581419363029	16.21238623498382	3.97927212826505
O	2.92475757007652	13.65561899209674	6.07371776987966

C	2.86736952472059	14.29859598180329	7.35977473810088
P	2.70442528379742	14.39147086598753	–2.11736630975029
C	1.04932828216295	14.20653899275622	–2.95547056594438
C	0.80103485476910	15.28333157067263	–4.01482434206063
C	–0.50603250793942	15.05836141858214	–4.78366391924553
C	–0.76837593975805	16.14338478563118	–5.82875308897921
C	2.63067452052227	13.12302713304451	–0.78786143074767
C	3.77742331998112	12.33250065691524	–0.60263494924779
C	3.80878481445966	11.33465921578578	0.37577227889157
C	2.68707291489634	11.11145232697991	1.17800235861544
C	1.53494158698959	11.88302915281092	0.99520307736540
C	1.50491558024263	12.87797576135347	0.01658749857048
H	3.87857625384588	14.53409274790949	7.72252741255952
H	2.39385579546266	13.57295793526547	8.02694129877499
H	2.25867926154182	15.21351068419272	7.31159472328082
H	3.09123945570991	12.61482653280530	3.77057392166777
H	4.05583115414754	13.65766018750904	1.75496030942245
H	3.99204051027006	16.17771851391508	6.05430023259927
H	4.95370477744719	17.21651147413752	4.05371432405875
H	5.12756326326240	18.35109723414641	2.06480259110361
H	5.97042395875138	18.72097309870273	–0.46767006300593
H	5.36756944512216	14.42668204317565	0.13077178393606
H	6.10794908522410	16.28630038887886	–1.67003053743989
H	3.16342000665259	17.41202667553648	–2.68579396891208
H	1.76294743786091	17.99809773973372	1.42362949920902
H	2.67787947532538	19.28295397128280	–0.78766394916627
H	2.70340484646564	10.32427365719229	1.93271236972004
H	4.70310849807896	10.72333641934137	0.50108834867516
H	4.64380063036415	12.48930412567475	–1.24931367246657
H	0.65178992983542	11.69955335329529	1.60849954813882
H	0.58954947245930	13.45365961805334	–0.12904452440215
H	1.70232337116552	15.34519855937827	0.86845141272216
H	–0.84077241510947	17.13694950796735	–5.36123216990294
H	–1.70772016993834	15.95960580860998	–6.36737355174451
H	0.04090077292620	16.18241955030756	–6.57299587082558
H	–0.47131506357051	14.06992580759337	–5.27069053249260
H	–1.34398804657100	15.01810244444858	–4.06778474886271
H	1.64507227295444	15.30849802001579	–4.72462235020806
H	0.76878776642574	16.27621517674861	–3.53525891614610
H	1.07403070441548	13.20736200460068	–3.41931368282811
H	0.23991863885184	14.20118419631468	–2.20914995450292

2c⁺ (planar)

E = –2726.308956680900 Eh

P	0.99406978659405	14.90242248702475	–0.20205494808614
C	0.64382351352929	16.59558021128539	–0.06105253387179
C	1.17803807057543	14.23070172780674	–1.87816046724645
C	1.13831010773942	13.80661453716255	1.16744042402109
C	0.52952527494845	17.41885153452494	1.12427087598101
C	0.36037560424580	18.77385029944425	0.69807012522945
C	0.43530952218705	18.81493708748719	–0.72649308655823
C	0.65054018957541	17.48878997978868	–1.20676958213506
Fe	2.16765356548377	18.02098155958155	0.06553321696856
C	3.73058136707952	19.06270616639706	–0.77593078343188
C	3.54573705563944	19.47545133657082	0.58077041020964
C	3.71190233231648	18.33194892962436	1.41842160087900
C	4.02764182105340	17.19687236031929	0.59734141532872
C	4.00842753965682	17.65930766125988	–0.77212318638759
C	4.26762481435536	15.83145299182462	1.05860485154942
C	4.83224031525182	14.86645977803904	0.20090468316705
C	5.10100540423925	13.57953038643196	0.64196850486853
C	4.82753329013292	13.21714913438841	1.97285732305061
C	4.26294629995589	14.16252265473944	2.84315806352998
C	3.98263809808933	15.44397233226013	2.37880973908836
O	5.13812078888007	11.94122490648252	2.31771756104655
C	5.05666242402159	11.58615196351134	3.70404160985515
C	2.59105024931023	14.29428485589309	–2.47675309222121
C	2.63998407944492	13.71769146701062	–3.89347194305394
C	4.04658294477691	13.77798971034238	–4.49073407785647
C	1.74079504112822	12.54736567025161	0.94983404014912
C	1.82376344369519	11.63176165170078	1.99308473941912
C	1.33212811187139	11.95866093366902	3.26112715024486
C	0.74520527932550	13.20992150015138	3.48231095979339
C	0.63584266552625	14.13058686307128	2.44621322128346
H	5.70983016863848	12.23091391067650	4.31160721918480
H	5.40064316216431	10.54907074451794	3.76445239837492
H	4.02142804789985	11.65532740398486	4.07234663382000
H	5.56011106588380	12.84108838692106	–0.01561598895730
H	5.10269791042841	15.14167239398540	–0.81812027569564
H	4.03088680436113	13.90414666848604	3.87387233379505
H	3.52901988251607	16.15816573706865	3.06664184907240
H	3.62088682701723	18.32729307902573	2.50007057161570
H	3.29370617056720	20.47706760939349	0.91624443688730

H	4.19237277371561	17.05344915323639	–1.65388246943770
H	3.65468796567661	19.69665076794839	–1.65433682100968
H	0.75500912906311	17.19469445805825	–2.24606230602506
H	0.22846549212148	19.62582958208104	1.35799937868640
H	0.37254404895195	19.70484328108668	–1.34529890788847
H	1.39051469121457	11.23368661052359	4.07311616387930
H	2.29007194595638	10.66286224864358	1.81678627566317
H	2.15611396666897	12.29227834902822	–0.02447765237994
H	0.34728440349855	13.46113845789152	4.46569033710795
H	0.13261618743950	15.07969636811876	2.61816183203141
H	0.53710575130799	17.07342642016710	2.15151371257472
H	4.40869508536496	14.81507259777296	–4.55319283270421
H	4.06574101292425	13.35810154759631	–5.50490613212167
H	4.76255992839279	13.20820971779040	–3.88018129732301
H	2.28585603198125	12.67368589109436	–3.87321984642784
H	1.93436823886383	14.26862193151348	–4.53739482217680
H	3.28805847967716	13.75028986071927	–1.82202884616292
H	2.93134157838974	15.34169113038563	–2.48589644802274
H	0.81245553034941	13.19319346097659	–1.82565554277491
H	0.45554971833618	14.77848555326297	–2.50341176839959

HPLC Chromatograms

1-[(R_P)-(*n*-Butyl)(phenyl)phosphine *P*-borane]-1'-(4-methoxyphenyl)ferrocene (1c)

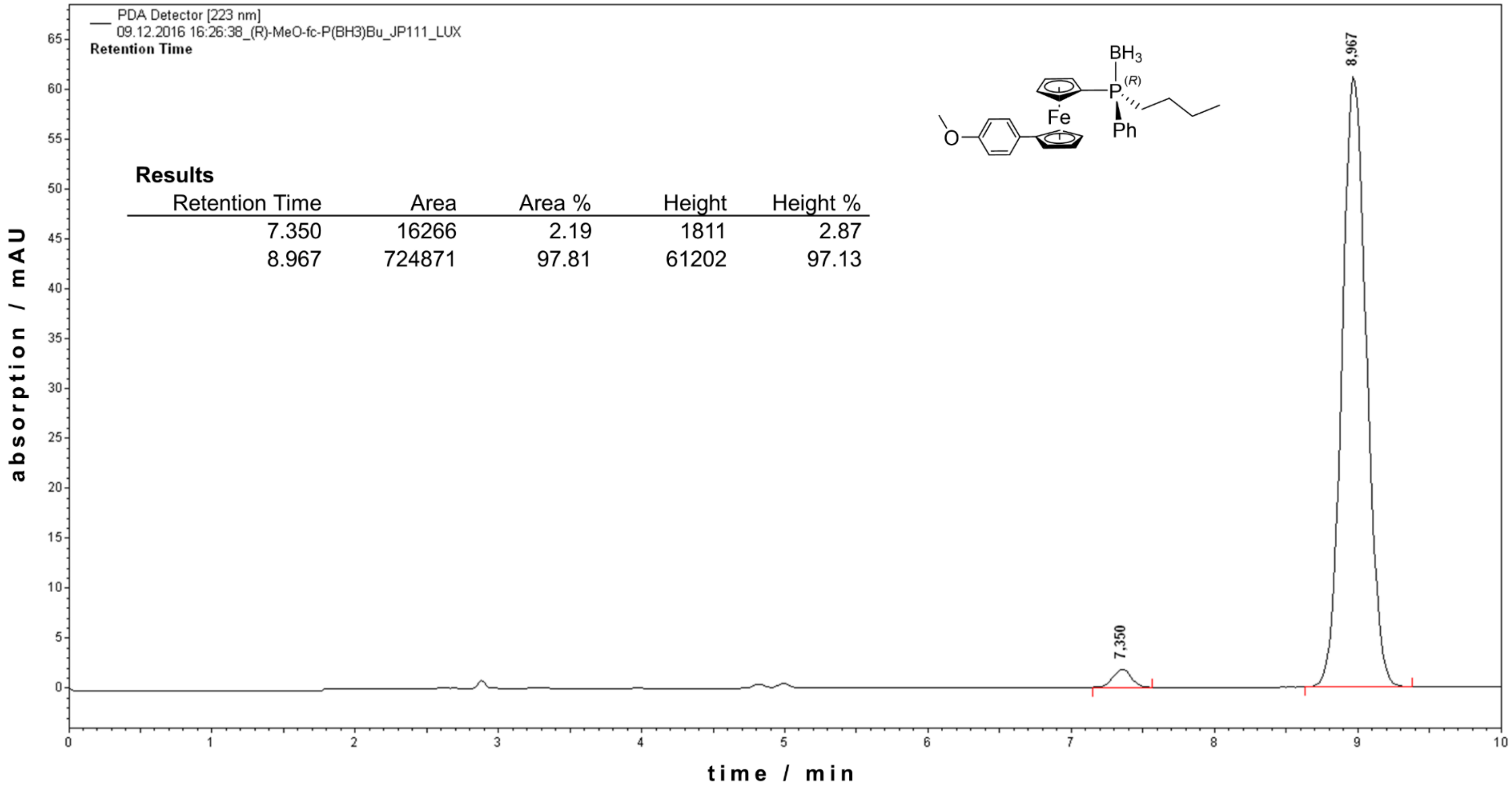

Figure S5. HPLC chromatogram of **1c**.

1-[(R_P)-(n-Butyl)(phenyl)phosphine]-1'-(4-methoxyphenyl)ferrocene (2c)*

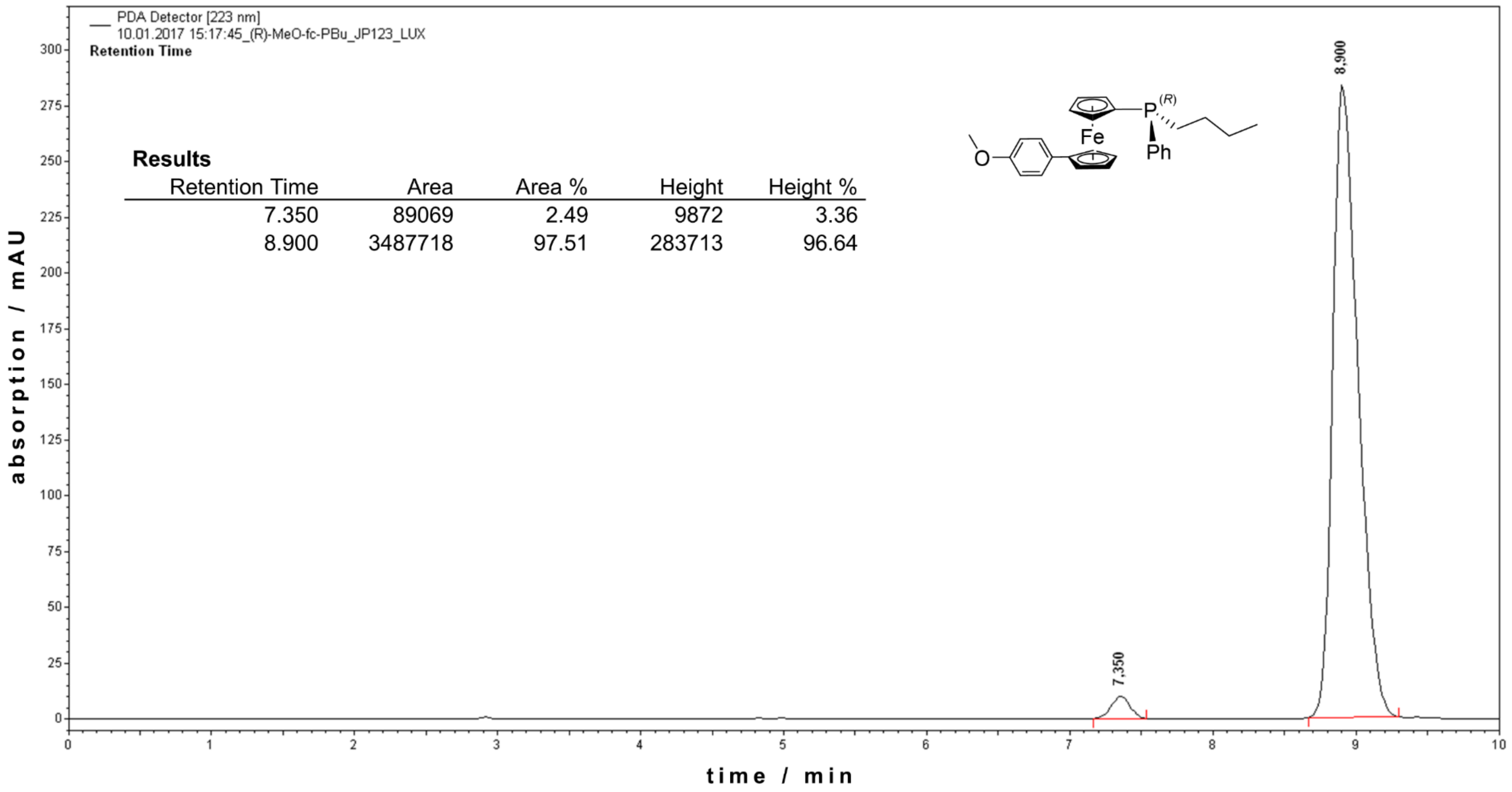

Figure S6. HPLC chromatogram of **2c**.

*Determined after reprotection by reaction with $BH_3 \cdot SMe_2$

NMR Spectra

1-[(R_P)-(*n*-Butyl)(phenyl)phosphine *P*-borane]-1'-(4-methoxyphenyl)ferrocene (1c)

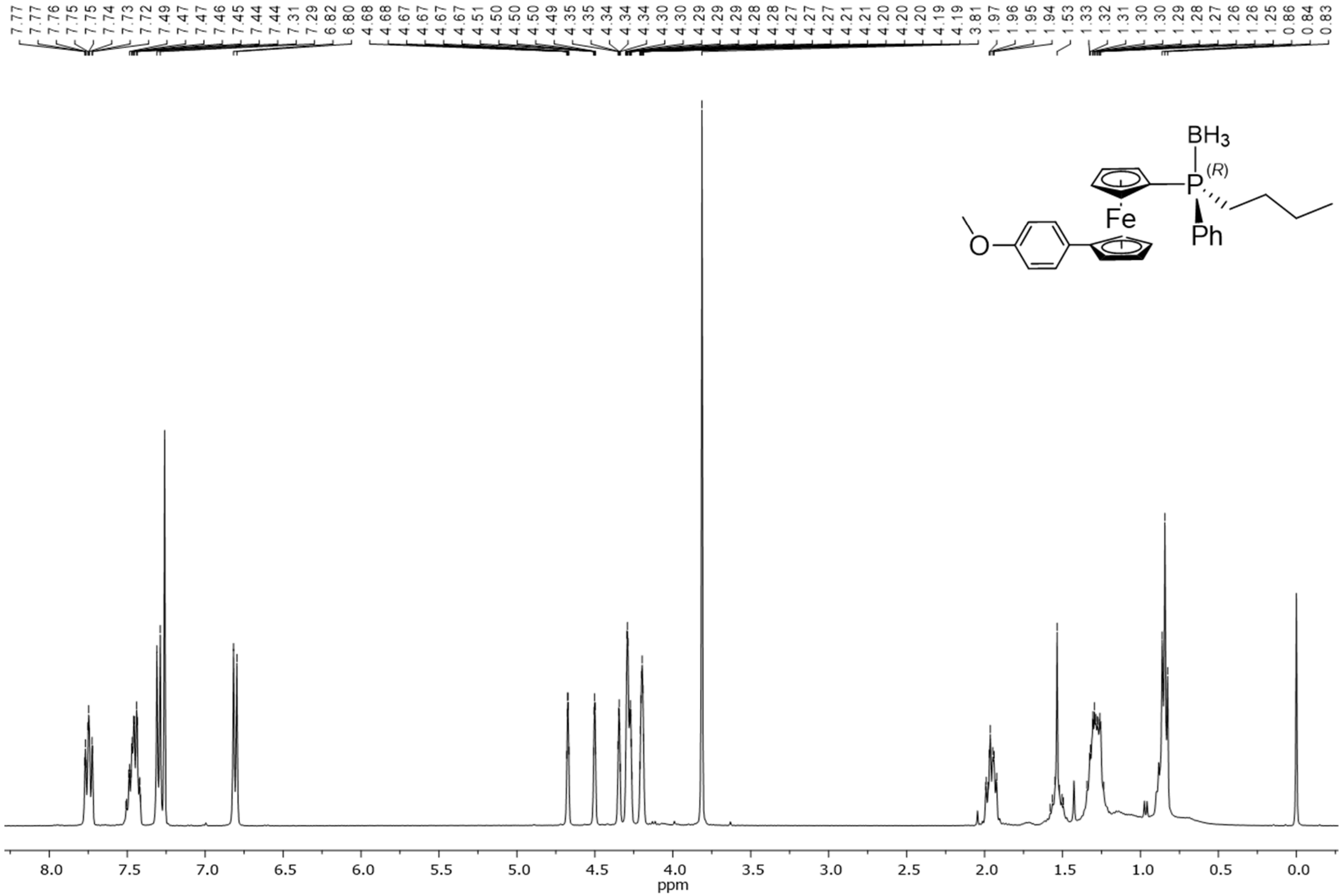

Figure S7. ^{1}H-NMR spectrum of **1c**.

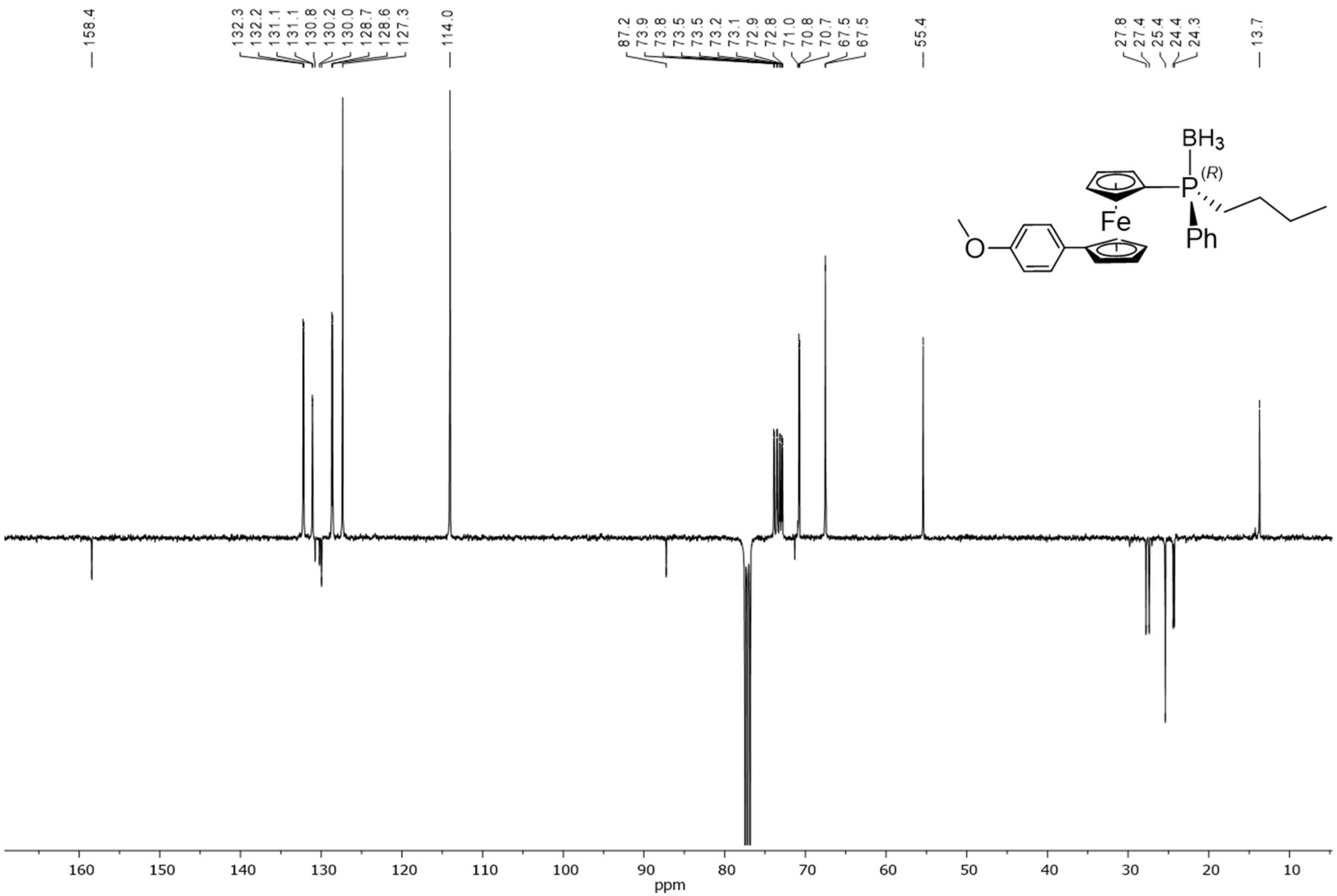

Figure S8. $^{13}C\{^{1}H\}$-NMR spectrum of **1c**.

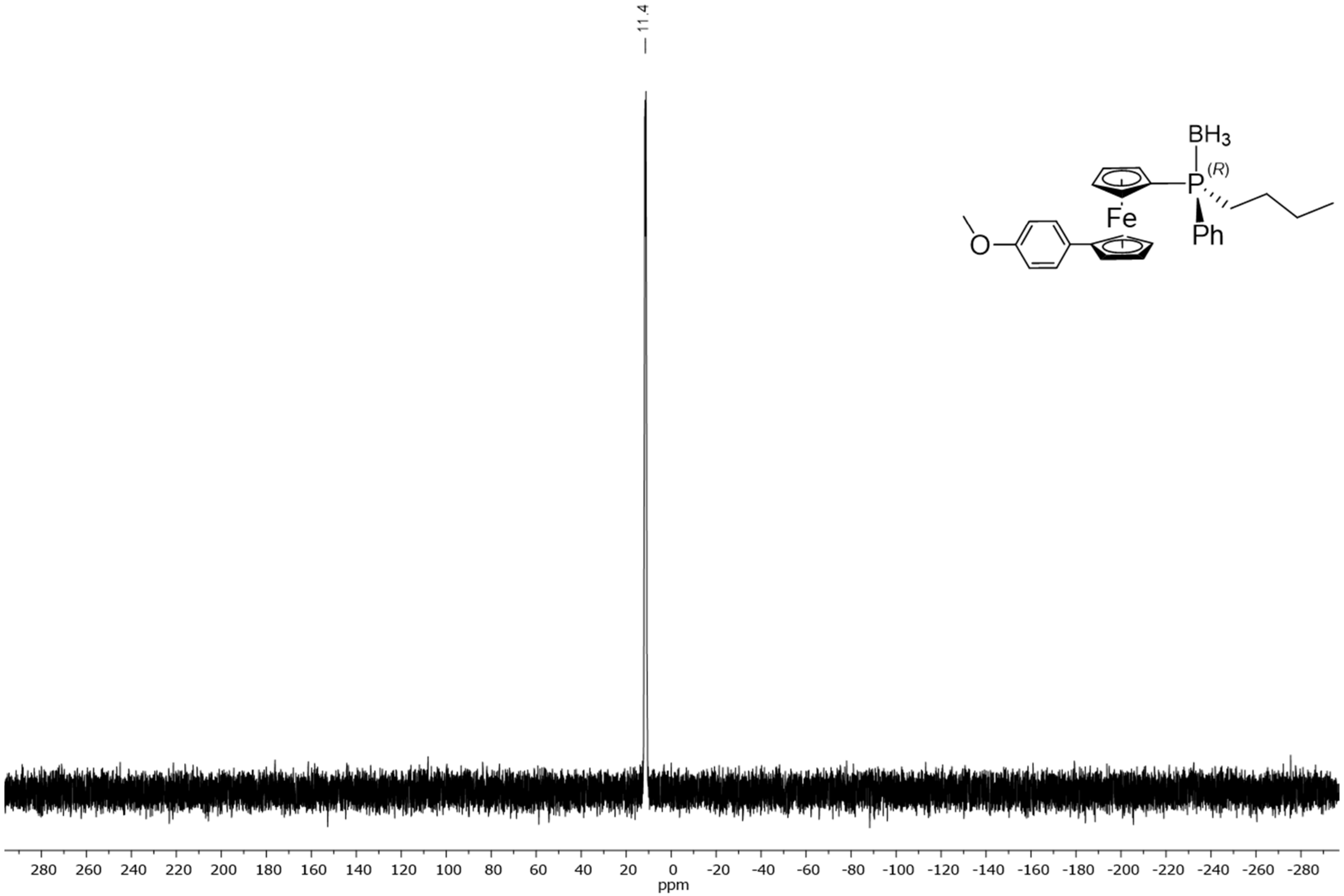

Figure S9. $^{31}P\{^{1}H\}$-NMR spectrum of **1c**.

1-[(R_P)-(*n*-Butyl)(phenyl)phosphine]-1'-(4-methoxyphenyl)ferrocene (2c)

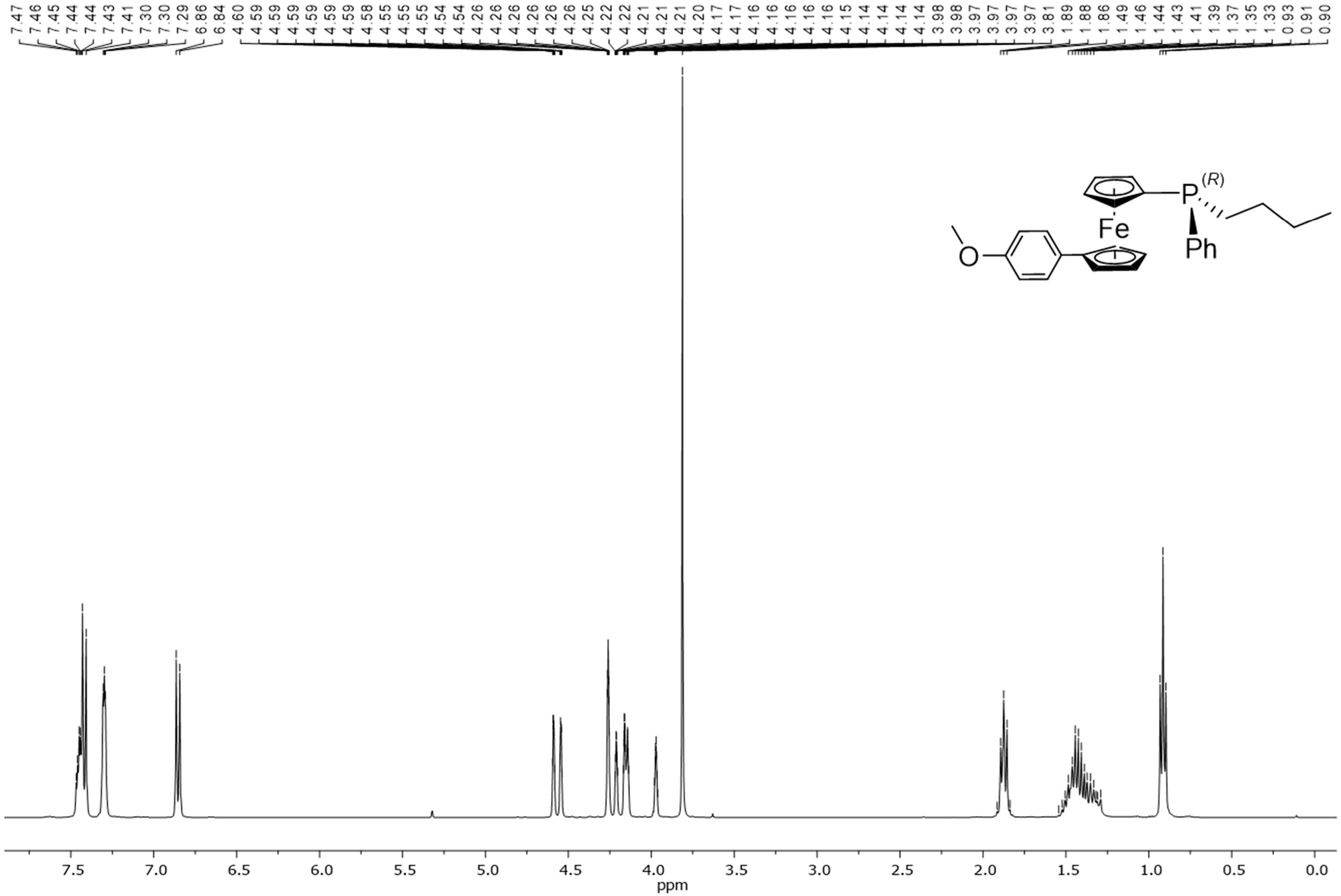

Figure S10. ^{1}H-NMR spectrum of **2c**.

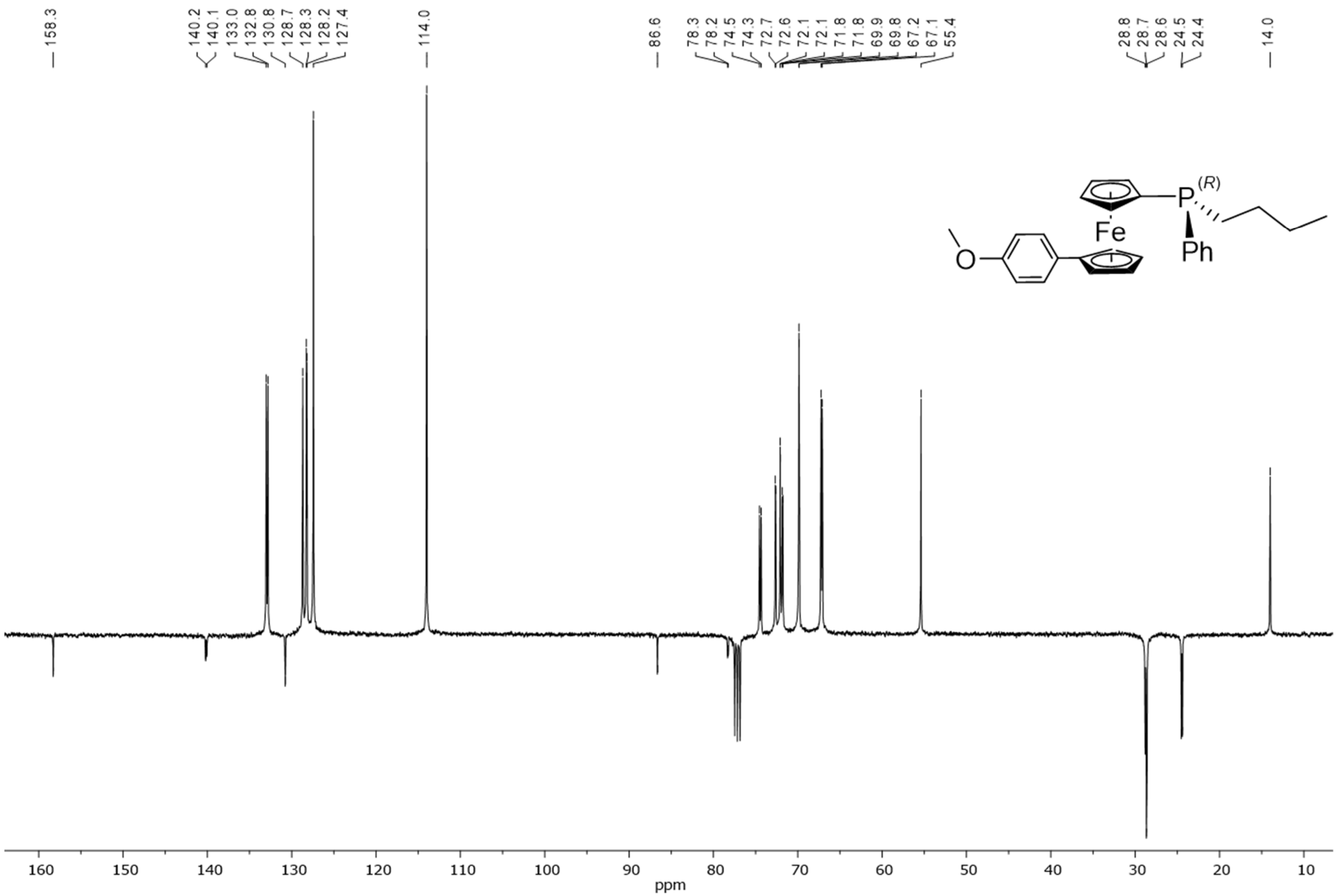

Figure S11. $^{13}C\{^{1}H\}$-NMR spectrum of **2c**.

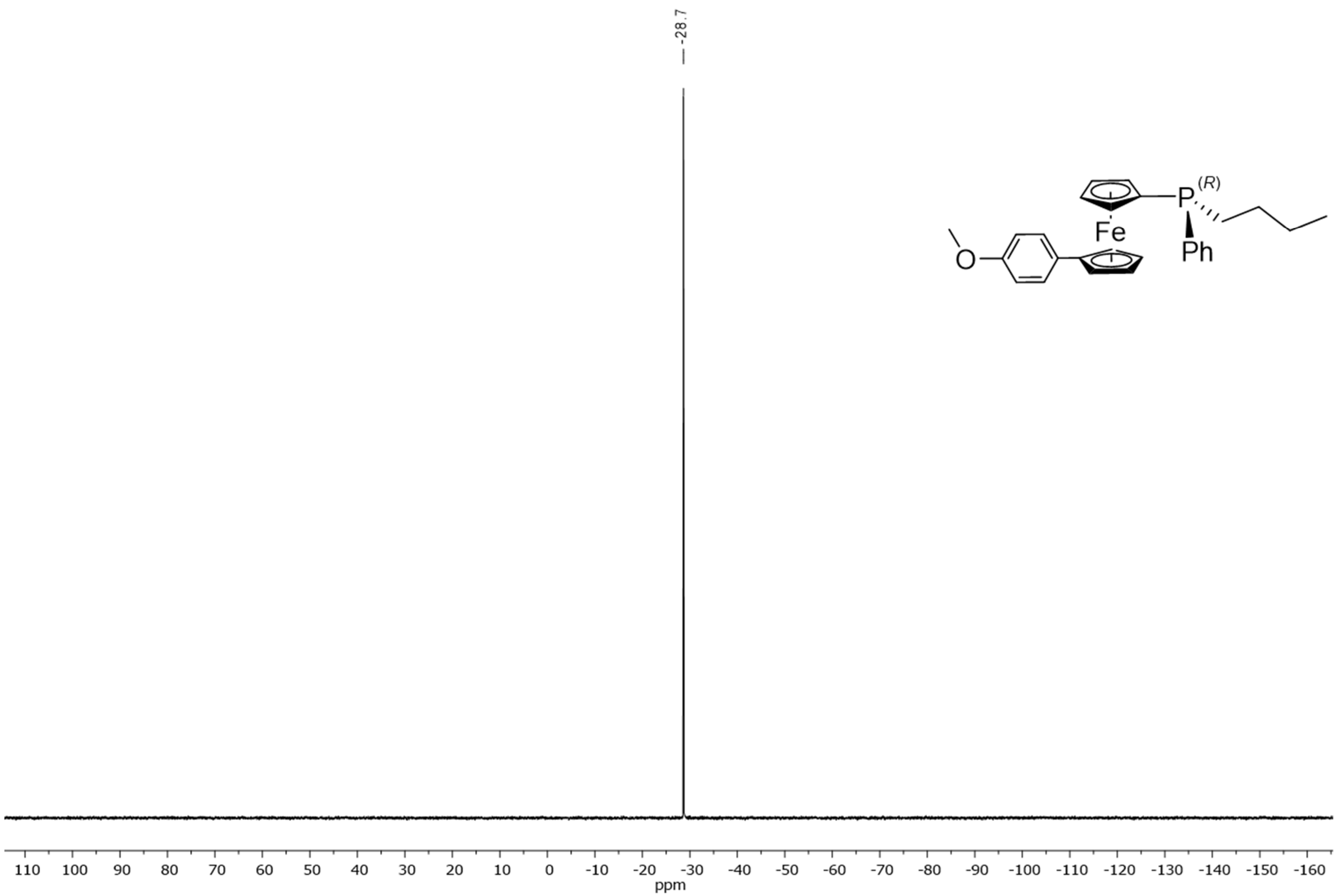

Figure S12. $^{31}P\{^1H\}$-NMR spectrum of **2c**.

1-[(R_P)-(*n*-Butyl)(phenyl)phosphino][(η^6-*p*-cymene)dichlororuthenium(II)]-1'-(4-methoxyphenyl)ferrocene (3c)

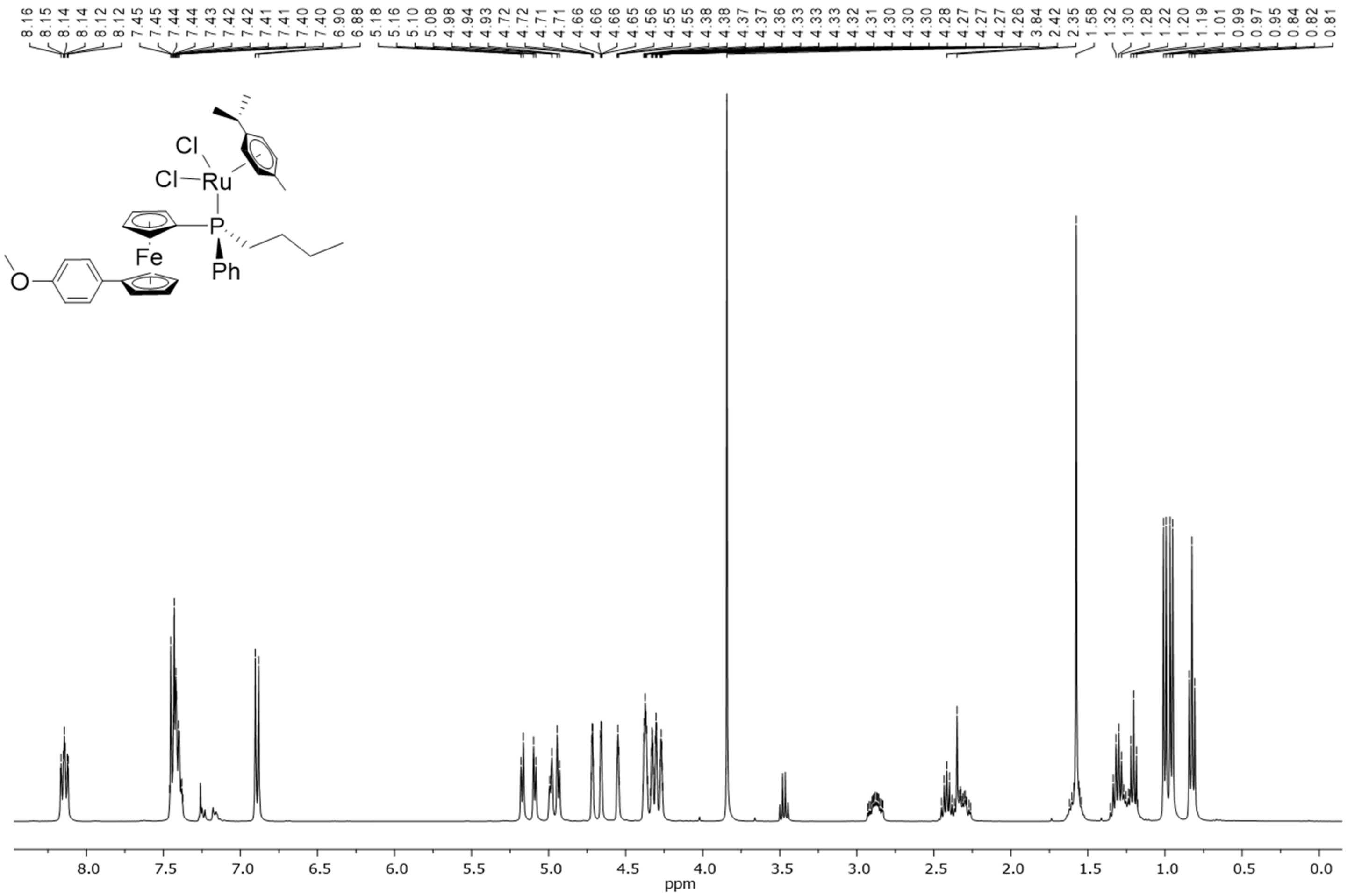

Figure S13. ^{1}H-NMR spectrum of **3c**.

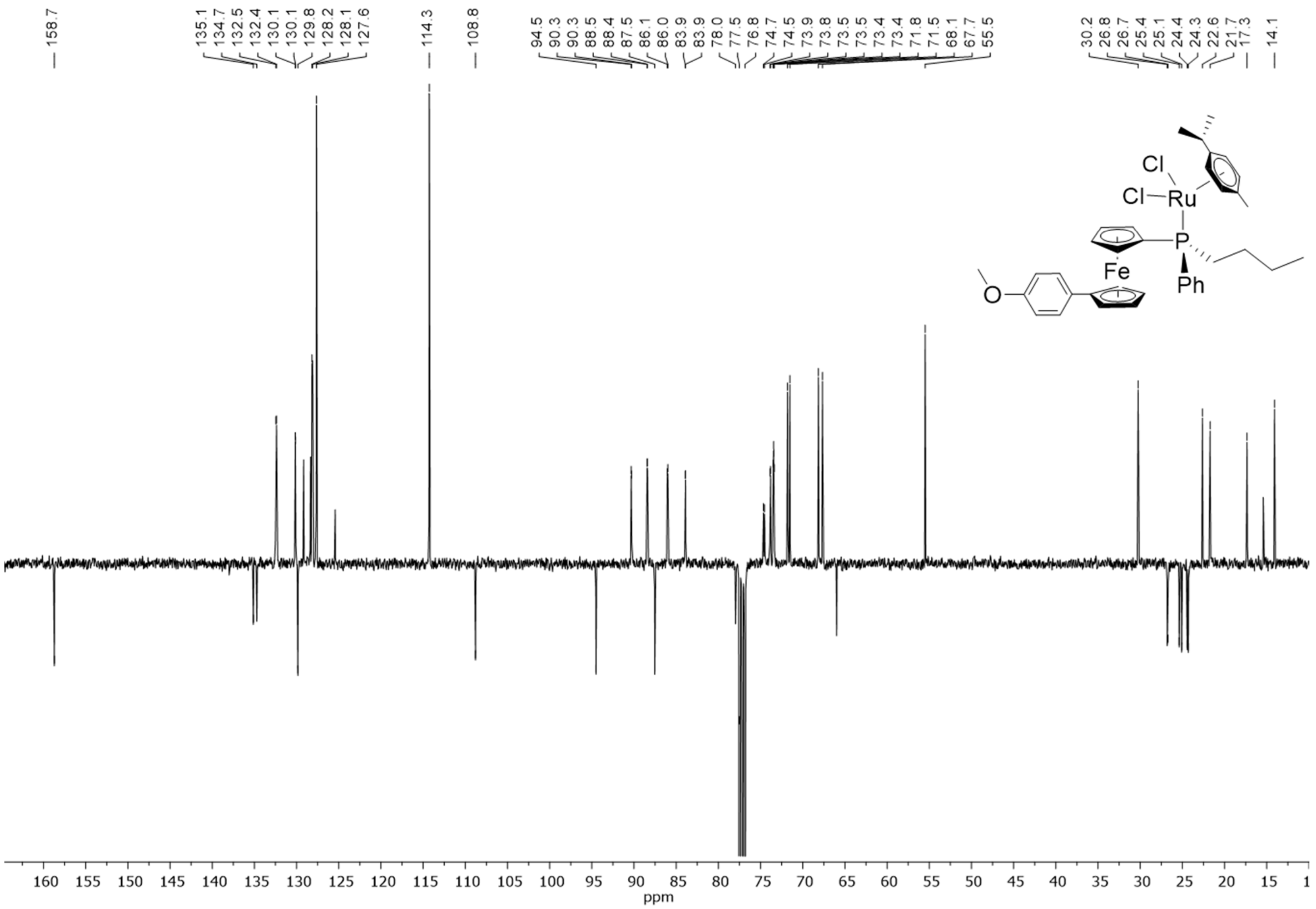

Figure S14. $^{13}C\{^{1}H\}$-NMR spectrum of **3c**.

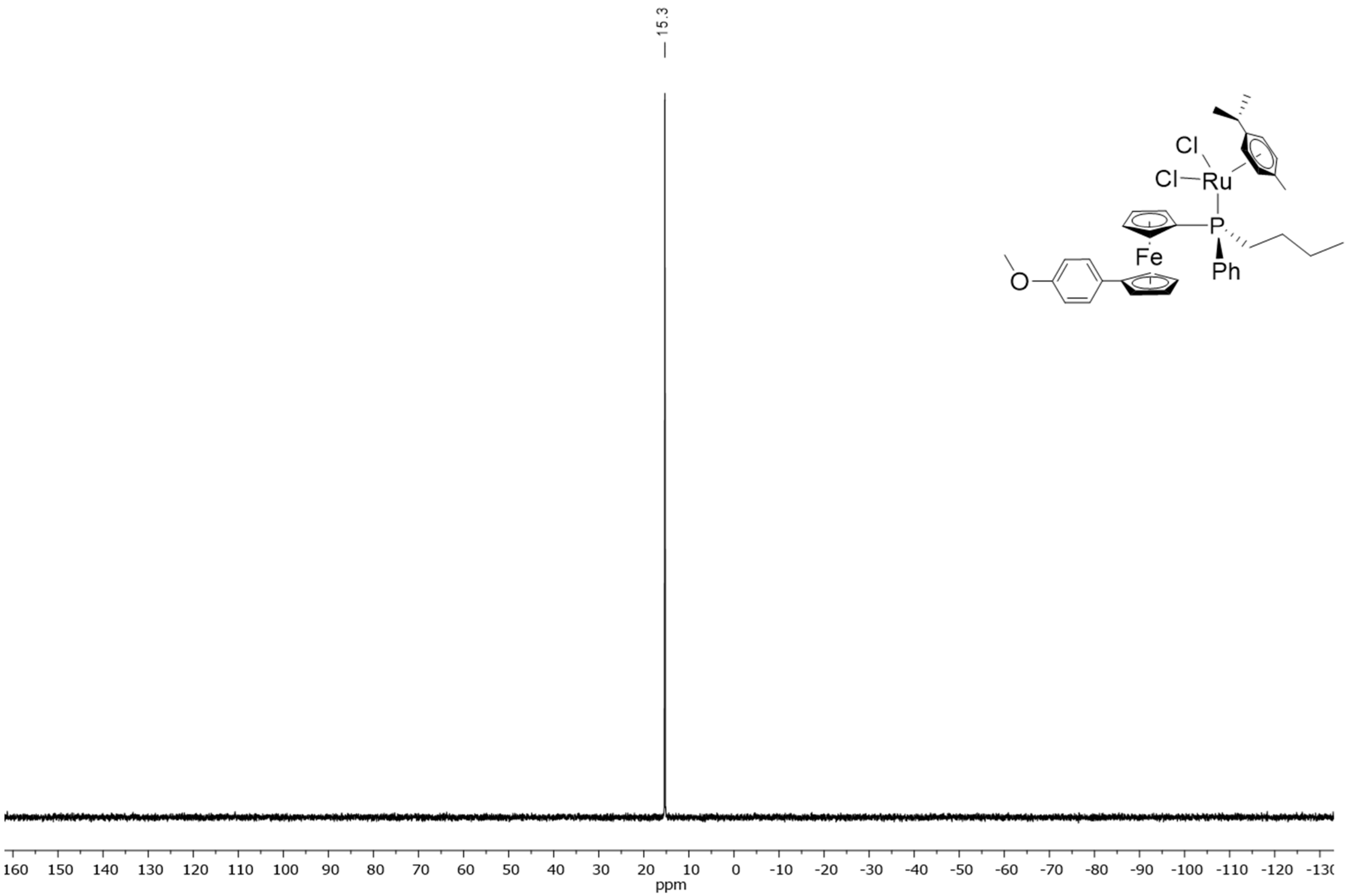

Figure S15. $^{31}P\{^{1}H\}$-NMR spectrum of **3c**

References

[1] P. Neumann, H. Dib, A.-M. Caminade, E. Hey-Hawkins, *Angew. Chem., Int. Ed.* **2015**, *54*, 311–314; *Angew. Chem.* **2015**, *127*, 316–319.

[2] M. S. Inkpen, S. Du, M. Driver, T. Albrecht, N. J. Long, *Dalt. Trans.* **2013**, *42*, 2813–2816.

[3] S. Jugé, M. Stephan, J. A. Laffitte, J. P. Genet, *Tetrahedron Lett.* **1990**, *31*, 6357–6360.

[4] J. Popp, S. Hanf, E. Hey-Hawkins, *ACS Omega* **2019**, *4*, 22540−22548.

[5] K. D. Reichl, D. H. Ess, A. T. Radosevich, *J. Am. Chem. Soc.* **2013**, *135*, 9354–9357.

[6] A. Rauk, L. C. Allen, K. Mislow, *Angew. Chem., Int. Ed.* **1970**, *9*, 400–414; *Angew. Chem.* **1970**, *82*, 453–468.

[7] R. D. Baechler, K. Mislow, *J. Am. Chem. Soc.* **1970**, *92*, 3090–3093.

[8] CrysAlis Pro: Data Collection and Data Reduction Software Package, Agilent Technologies.

[9] SCALE3 ABSPACK: Empirical Absorption Correction Using Spherical Harmonics.

[10] G. M. Sheldrick, *Acta Crystallogr., Sect. A Found. Crystallogr.* **2008**, *64*, 112–122.

[11] C. F. Macrae, P. R. Edgington, P. McCabe, E. Pidcock, G. P. Shields, R. Taylor, M. Towler, J. van de Streek, *J. Appl. Crystallogr.* **2006**, *39*, 453–457.

[12] C. F. Macrae, I. J. Bruno, J. A. Chisholm, P. R. Edgington, P. McCabe, E. Pidcock, L. Rodriguez-Monge, R. Taylor, J. van de Streek, P. A. Wood, *J. Appl. Crystallogr.* **2008**, *41*, 466–470.

[13] F. Neese, *Wiley Interdiscip. Rev. Comput. Mol. Sci.* **2018**, *8:e1327*.

[14] F. Neese, *Wiley Interdiscip. Rev. Comput. Mol. Sci.* **2012**, *2*, 73–78.

[15] A. D. Becke, *Phys. Rev. A* **1988**, *38*, 3098–3100.

[16] J. P. Perdew, *Phys. Rev. B* **1986**, *33*, 8822–8824.

[17] J. P. Perdew, *Phys. Rev. B* **1986**, *34*, 7406–7406.

[18] F. Weigend, R. Ahlrichs, *Phys. Chem. Chem. Phys.* **2005**, *7*, 3297.

[19] F. Weigend, *Phys. Chem. Chem. Phys.* **2006**, *8*, 1057.

[20] L. Goerigk, S. Grimme, *J. Chem. Theory Comput.* **2011**, *7*, 291–309.

[21] L. Goerigk, A. Hansen, C. Bauer, S. Ehrlich, A. Najibi, S. Grimme, *Phys. Chem. Chem. Phys.* **2017**, *19*, 32184–32215.

[22] L. Goerigk, S. Grimme, *Phys. Chem. Chem. Phys.* **2011**, *13*, 6670.

[23] S. Grimme, S. Ehrlich, L. Goerigk, *J. Comput. Chem.* **2011**, *32*, 1456–1465.

[24] S. Grimme, J. Antony, S. Ehrlich, H. Krieg, *J. Chem. Phys.* **2010**, *132*, 154104.

[25] F. Neese, F. Wennmohs, A. Hansen, U. Becker, *Chem. Phys.* **2009**, *356*, 98–109.
